# Long-term trends in the honeybee ‘whooping signal’ revealed by automated detection

**DOI:** 10.1371/journal.pone.0171162

**Published:** 2017-02-08

**Authors:** Michael Ramsey, Martin Bencsik, Michael I. Newton

**Affiliations:** Department of Physics and Mathematics, Nottingham Trent University, School of Science and Technology, Clifton Lane, Nottingham, United Kingdom; University of Arizona, UNITED STATES

## Abstract

It is known that honeybees use vibrational communication pathways to transfer information. One honeybee signal that has been previously investigated is the short vibrational pulse named the ‘stop signal’, because its inhibitory effect is generally the most accepted interpretation. The present study demonstrates long term (over 9 months) automated *in-situ* non-invasive monitoring of a honeybee vibrational pulse with the same characteristics of what has previously been described as a stop signal using ultra-sensitive accelerometers embedded in the honeycomb located at the heart of honeybee colonies. We show that the signal is very common and highly repeatable, occurring mainly at night with a distinct decrease in instances towards midday, and that it can be elicited *en masse* from bees following the gentle shaking or knocking of their hive with distinct evidence of habituation. The results of our study suggest that this vibrational pulse is generated under many different circumstances, thereby unifying previous publication’s conflicting definitions, and we demonstrate that this pulse can be generated in response to a surprise stimulus. This work suggests that, using an artificial stimulus and monitoring the changes in the features of this signal could provide a sensitive tool to assess colony status.

## Introduction

Honeybee *(Apis mellifera)* colonies kept for commercial purposes are usually housed in beehives, with population sizes exceeding 40,000 individuals depending on the season. During the spring/summer active season there is a higher volume of workers assigned to gathering resources, such as pollen and nectar, to sustain them over the inactive winter months when the population size reduces [1]. Colonies are comprised of a single queen, a few hundred male drones and thousands of female worker bees, as well as developing eggs, larvae and pupae within the honeycomb [1,2].

Within the context of food processing, worker bees have numerous day to day tasks distributed based on the age of the bee [3]. Older bees (around 30% of the colony) leave the hive to forage whilst the youngest of the workers stay in the hive, processing the pollen and nectar as it arrives (reviewed by Robinson [4]). Honeybees are therefore very structured in their operations, communicating via numerous signal pathways to coordinate their activities [2]. Honeybees will naturally seek out dark areas to build their nests, such as tree cavities [5], and within the dark confines of a beehive, visual communication is almost impossible. In light of this, recent focus has been on how they communicate using substrate-borne vibrations.

Our study focuses on the long term monitoring and the resulting statistics of a type of honeybee vibrational pulse recently named in the literature as the ‘stop signal’, which is one of three established types of worker piping. Regardless of the type, worker piping is thought to be generated by a bee that contracts her thoracic muscles whilst in contact with the honey comb. This ‘stop signal’ is believed to be transmitted through the sender striking its head into a recipient whilst pressing her thorax against the honeycomb [6] and producing a relatively brief vibrational pulse [7] with her wing muscles. This signal has been observed at frequencies of between 200-400Hz for a duration of 0.05–0.2 seconds [8, 9, 10]. Having a mean duration of 0.14 seconds and negligible inherent frequency sweep [9], it differs significantly from the other, much longer, forms of worker piping [11, 12, 13]. These other two, wings together and wings apart piping, have a much longer duration within 0.5–2 seconds [8, 14, 15, 16] with the former exhibiting a frequency sweep upwards from 100-200Hz [16]. Seeley and Tautz [16] identified wings together piping by nest scouts acts to stimulate other bees to warm up their wing muscles prior to lift-off during preparation for swarming. Wings apart piping, however, can be found under many other circumstances within the hive [8, 14, 15, 16].

There is some controversy in the literature over the function of the brief vibrational pulse of main interest to our present study. It was first described by Esch [11] as a begging call for trophallaxis by a dance follower to a waggle dancer advertising a nectar source. According to this hypothesis, the piping bee is requesting a sample of the nectar brought home by the dancer. This ‘begging call’ hypothesis also features in an earlier study by Von Frisch [17, 18] in which he examined how the bees following a dancer gain knowledge of the scent of the flowers that the dancer is advertising. “The signal may cause the dancer to interrupt, allowing the follower to approach the dancer for food” [17]. Esch [11] replayed sounds recorded on tape into the hive and observed that all the bees became motionless if the sound was of very high intensity or was transmitted through the substrate. The phenomenon of immobilising honeybees using various types of strong vibrations has also been explored by several groups [19, 20].

The ‘begging signal’ hypothesis features prominently throughout Michelsen’s [6, 12, 21] studies and was generally accepted until Nieh [7] presented an alternative hypothesis for the function of this pulse: it serves as a stop signal to prevent foraging at overexploited food sources by inhibiting recruitment by waggle dancing bees [8, 22]. This stop signal hypothesis arose from Nieh’s [7] observation (later supported by Thom *et al*. [13]) that the primary senders of these pulses are nectar foragers that are engaged in performing tremble dances. Kirchner [8] stated that there are vibrational signals emitted by tremble dancers, supporting the hypothesis that there is a vibratory channel of communication employed within tremble dances and that these are indistinguishable from those produced by the dance followers recorded by Michelsen *et al*. [6]. Allowing a large number of foragers to build up at a feeder has several effects such as increasing forager wait time and colony nectar intake. Lau and Nieh [9] recorded 100% more of these pulses from bees returning from a crowded feeder as compared to a feeder at which they did not need to wait.

Kirchner [8] demonstrated that waggle dancers that received a stop signal had shorter dance durations and were more likely to leave the dancefloor but also that dancers did not show a strong, freezing response to a stop-signal [7, 10, 22], nor did they then offer nectar to their pipers. This is characteristic of modulatory signals, which are produced in a variety of contexts and are characterized by slightly shifting the probability of receiver behaviours, depending upon receiver response thresholds [7,13]. Producing a modulatory signal makes perfect functional sense under the condition of a colony experiencing an excessive surge in its nectar influx, so that the colony needs to supress the recruitment of additional nectar foragers and facilitate the recruitment of additional nectar receivers [13]. Inhibiting waggle dancers can also have a secondary effect in that more nectar receivers can be recruited (reviewed in Keitzman and Visscher [23]). Thus it is not surprising to see that tremble dancers act to inhibit waggle dancers, as reported by Lau and Nieh [9], Nieh [7] and Thom *et al*. [13].

The brief vibrational pulse investigated in our present study has also been identified as having another function: a signal for danger [24]. Nieh [25] suggested that a bee may produce this signal upon return to the hive in response to a traumatic experience she received at a food source. This is hypothesised to be a method of preventing a particular location being advertised to prevent other bees from suffering a similar fate. In the study Nieh [25] pinched the leg of a bee at the food source to replicate an insect bite and recorded these signals upon return. Interestingly, the study showed that a bee who came under attack and won the battle or came back unharmed did not signal danger. A recent study by Tan *et al*. [26] using tethered hornets also showed a great increase in these signals on waggle dancers who are advertising food sources which had a predator present. Interestingly it was shown that the larger the hornet, the greater the number of these pulses that were recorded [26]. They also showed that a honeybee will identify the dangerous location that is being advertised based on the smell of the dancer [26].

Aside from its function within the context of foraging, Seeley *et al*. [27] presented an excellent study showing that this pulse played an integral role within the democracy of choosing a nest site during swarming via a process called “cross-inhibition”. Waggle dancers advertised the locations of their preferred nest sites and scout bees used ‘stop signals’, identified visually through a head-butt twinned with microphone recordings, to inhibit the dancers of locations that they deemed less favourable [27].

Nieh [7] also suggested that, although these pulses may act to influence other bees, such as food-storers or nurse bees, waggle dancers do appear to be the main focus. Through playbacks of the signal via a vibration probe, waggle dancers significantly reduced the amount of time that they danced for [7]. It was also shown that most of the foragers did not stop immediately during or after receiving a 150ms vibration, so this inhibitory effect on waggle dancers cannot be explained by a general freezing response to vibrations [7].

For clarity purposes, for the remainder of this paper we will refer to this pulse as the honeybee “whooping” signal as this is onomatopoeic of the produced vibration. Alternative terminology such as “begging signal” emphasise a possible singular function of the signal made by dance followers, which seems questionable after the recent work of Nieh [7]. ‘Stop signal‘ emphasises that it is an inhibitory signal produced mainly to stop waggle dancers from advertising potential nest sites and food sources under a variety of different contexts.

In spite of current literature, our own observations (see Supplementary Videos) suggests that this pulse isn’t restricted to its current definition of ‘stop signal’. In fact, (probably accidental) collisions between bees can often result in the detection of a pulse indistinguishable to what has been described as a honeybee stop signal, even though there is no waggle dance or trophallaxis involved. Numerous other videos of ours show that it is very rare to find any visual evidence of this pulse taking place even though they can be heard very frequently, many times in one minute. In addition to this, it is easy to demonstrate that upon gentle shaking (or even if you were to knock gently on a hive) this pulse is generated en masse by the bees. It is therefore the aim of this study to create and optimise software that can accurately explore months of continuous vibrational data recorded non-invasively from the heart of honey bee colonies, collecting the timings of specific honeybee pulses of interest. Discussion of the resulting statistics on these pulses helps in suggesting that what has recently been systematically described in the literature as a honeybee stop signal is actually a remarkably common signal, being produced under many different circumstances.

The outcomes of this study also allows us to 1) discuss results in terms of any evidence of long term trends; 2) discuss results in the context of the colony status; 3) compare results with stop signals measured from within two different locations of the same frame; 4) discuss the results measured from a colony in the UK and another one in France and; 5) explore the possible correlations between ‘whooping’ signal occurrences and weather trends, as these will strongly modulate some colony responses, in particular in terms of foraging and thermo-regulating activities. It is expected, for example, that weather patterns that would inhibit foraging would cause a decrease in waggle dances thus reduce the need for the associated ‘stop signals’.

## Methodology

No ethical approval was required as this study wholly focussed on the *in-situ*, non-invasive, acquisition of data from invertebrate colonies.

### Vibrational measurements

The recordings of vibrational data were taken from 2 hives, one located at the Clifton campus of the Nottingham Trent University (NTU), UK and the other at an apiary in Jarnioux, France after being granted specific permission from NTU Estates and Mr Joseph Bencsik respectively.

The UK Hive was monitored for around 4 months commencing on the 29/07/2014 until termination on the 11/11/2014, allowing us to monitor a colony as they prepare for winter. The French hive has been continuously monitored from 16/04/2015 (and is still ongoing) but our analysis only considers data until 24/12/2015, disclosing the evolution of a colony across the entire active season. The data were analysed separately for each hive to allow comparison of data taken from two different climates.

**Fig 1:**
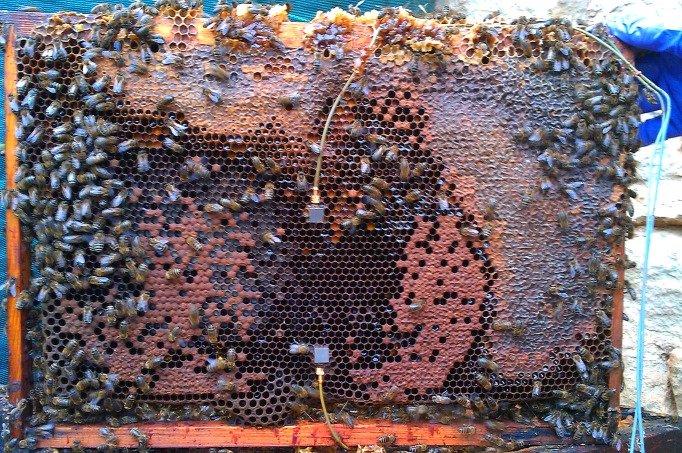
Accelerometer configuration. A brood frame equipped with accelerometers in the centre and at the periphery, located in the centre of the hive. This photo was taken one year after installation, with accelerometers surrounded by honeycomb cells in near perfect condition. The photo shows the French set-up which is identical to the UK one.

The recording of vibrational data was achieved using two ultra-high performance accelerometers (Brüel and Kjær, 1000 mV/g) placed in the middle of the honeycomb along a vertical line, one in the centre, and the other 7cm lower down (Fig 1). At the time of installation, small amounts of molten wax were dripped into the accelerometers to secure them onto the frames and avoid the exposure of metal components. This frame was then placed back into the centre of the brood box. The accelerometers were polarised with individual ENDEVCO 4416B conditioners (MEGGITT, U.S.A.), the output of which was plugged into an i02 sound card (ALESIS, U.S.A.) for digitisation at 22kHz sampling rate. Vibrational data sets were continuous and made up of one-hour long audio files, stored on an external 4TB storage station via a home-built bash code on a Linux O.S. based computer that was set to reboot itself every 100 hours, with recordings resuming automatically upon rebooting or power loss and auto-recovery.

Software was then written in MATLAB® (The Mathworks, USA) and algorithms were optimised to detect and record the exact times at which whooping signals occurred within the vibrational data sets (See S1 File for more information). The whooping signal detection is a two-step process. First, a spectrogram of a template pulse signal is matched to a spectrogram of the continuous recording by the ratio of the cross correlation product and the Euclidean distance (See S1 and S2 Figs). In this way, whooping signals and other pulses are detected. Second, the detected pulses are discriminated into ‘whooping signals’ and 'non-whooping signals’ by simple discriminant function analysis on PCA scores. When data sets consisting of the exact timings of the genuine-only honeybee whooping signals that occurred within each vibrational data set were identified, these were used to show the quantitative statistics of their occurrences over the entirety of both the UK and the French data sets.

### Whooping signal parameter analysis

All data analysis, statistical and graphical, was undertaken in MATLAB® with occasional use of the statistics toolbox. All data was tested for normality using the Kolmogorov-Smirnov test and in some instances normalisation is undertaken using Log_10_ transformation. When normal distribution cannot be achieved, the non-parametric equivalent test is used. A Generalised Linear Model is used for the analysis of time on both frequency and amplitude, and the Spearman’s rank Correlation is used to test the relationship between frequency and amplitude of whooping signals.

### The brood cycle and daily average

To see how the occurrences of whooping signals changed throughout the course of an average day, the number of whooping signals that occurred at each hour of the day is averaged and standard error is also shown on Fig 3 for the French data, and S4 Fig for the UK data. To examine the effect of the brood cycle, the daily modal amplitude of vibrations as a whole is explored as part of Fig 9.

### The effect of weather on the occurrences of whooping signals

UK weather data was obtained via an onsite TechnoLine WS-2350 weather station. The French weather data was supplied free of charge by Météo France (www.metofrance.com) for the site and dates that we required. Outside temperature, outside humidity and rainfall were analysed with the addition of atmospheric pressure in the UK dataset.

### Analysis of duplications

First, the raw data of whooping signal timings is uploaded for both accelerometers on one frame. For any whooping signal found on the first accelerometer, the nearest whooping signal, in time, is found on the second accelerometer and the difference inspected. If its absolute value is smaller than 10ms, then the pulse timing is labelled as a duplicated. From this, the percentage of duplications can be calculated by dividing the number of ‘duplicates’ by the total number of whooping signals, for example over one hour. The outcome of this processing is shown in Fig 9. The brood cycle daily modal values were then superimposed over the percentage of duplications to help visualise the effect of honeycomb mass-density on signal duplications. This also allowed to us to estimate the range of detection for the accelerometers.

### Video recordings

Two accelerometers were also secured on the central frame of a separate colony kept in our laboratory, in Nottingham, UK, in a standard brood box. A transparent Perspex cavity was placed on the top of the hive, allowing this frame to be occasionally extracted and observed with video analysis on both sides, with the soundtrack provided by the accelerometer signals, for no longer than 20 minutes every day, up to twice a week, whilst being replaced in the brood box for the majority of the time. This allowed this colony and this frame to develop naturally without the major disturbances usually associated with permanent observation hives, where bees are forced to live in a planar geometry. Some excerpts of these videos have been included to show examples of other instances in which whooping signals can occur within the hive and aid in the discussion of the statistics of these signals generated as part of this study.

## Results

Analysis of the whooping signal dataset recorded in France is presented below. For comparison, the analysis of the UK dataset is supplied in S3–S8 Figs of the Supplementary Material.

### The physical characterisation of the ‘whooping signal’

**Fig 2:**
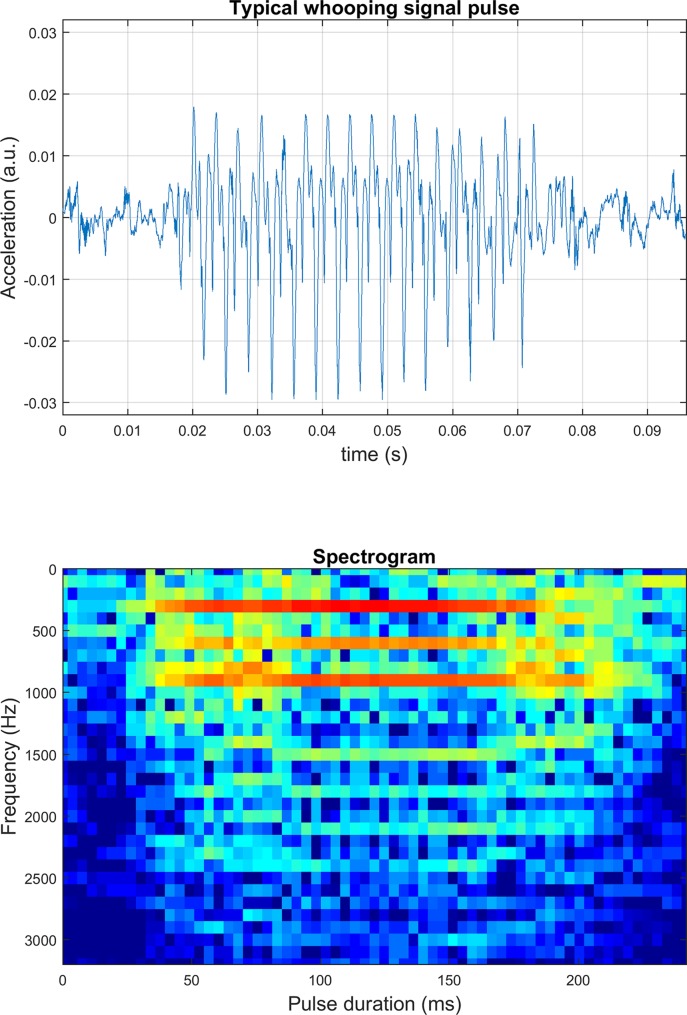
The time course and corresponding spectrogram of a typical honeybee whooping signal. The colour intensity of the spectrogram denotes the logarithmic amplitude of the measured acceleration with red being the highest acceleration and dark blue being the lowest.

The time course of the acceleration and the frequency components for a typical honey bee whooping signal, measured with an accelerometer, is shown in Fig 2. This particular signal has a fundamental frequency of 355Hz for a duration of 60ms. A common feature of this signal is to have two well pronounced upper harmonics at twice and three times the value of the fundamental frequency. Numerous harmonics at even higher frequencies can also be seen, with negligible but measurable amplitudes. Another common component of the signal that can be seen on both the time-course and the spectrogram is the ultra-short increase and decrease in the oscillation’s frequency respectively at the start and the end of the pulse. Our spectrograms agree remarkably well with that of the template “begging signal” published by Michelsen [21], which is provided in S9 and S10 Figs. In S1 Movie, the sound, waveform, spectrum and spectrogram of hundreds of randomly chosen whooping signals displays a sample of the exact pulses that were processed by our software. Although we cannot show the entire collection of pulses that have been detected, our limited tests strongly support the fact that the vast majority of pulses that we have analysed are exclusively “whooping signals” as understood by experts.

**Fig 3:**
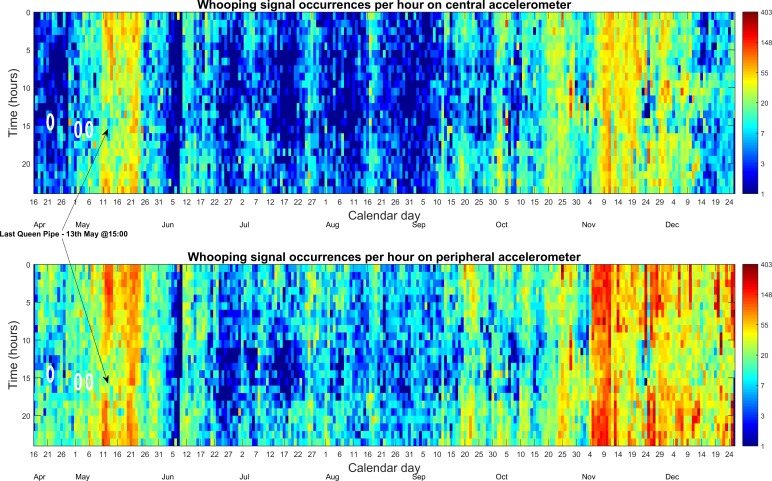
Whooping signal hourly occurrences. Central (top) and peripheral (bottom) accelerometer logs of the French hive (2015 season). The colour codes the number of hourly occurrences from dark blue (≤1) to dark red (403 signals) on a logarithmic scale. White circles highlight the occurrences of the three swarms that occurred from this hive, with the first one being the primary swarm.

### Long-term statistics of whooping signals from within the French hive

**Fig 4:**
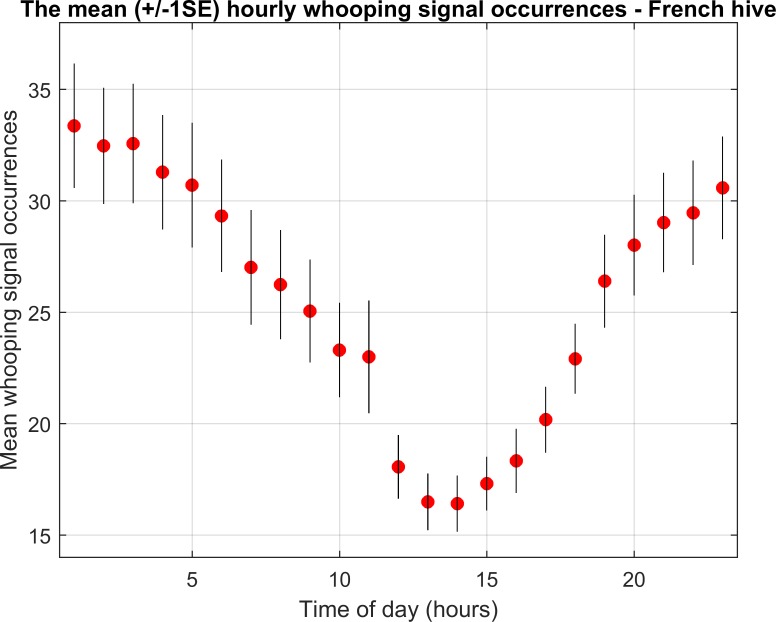
Average number of whooping signal occurrences observed for each hour of the day. This graph is obtained over the vibrational dataset shown in the previous figure. The vertical bars indicate +/-1 standard error (SE).

Fig 3 clearly demonstrates that (i) the signal occurs very frequently (up to 6–7 times per minute), and that (ii) there is a pronounced and consistent midday decrease of occurrences, further evident upon averaging the hourly whooping signal occurrences for all 261 days (Fig 4). It is seen that there is a large increase in occurrences after the last swarm and during the winter months. It is also seen that detections are modulated by the brood cycle with peaks every 21–24 days. Similar trends can be seen in the UK data although on a shorter time scale (see S3 and S4 Figs). S1 Audio, recorded between midnight and 1 am on 12^th^ May 2015, corresponds to a hotspot on Fig 3. Amongst other signals, including queen tooting, (that were successfully dismissed by our software), it is easy to check by critical listening, the genuine commonness of whooping signals occurring during this time and also how different they are compared to the various other signals. For comparison, in S2 Audio we have provided a sample of the “wings together” and “wings apart” pipes [8, 14, 15, 16] that were filtered out by our software at the “second pass” of the detection process.

Fig 4 shows that there is a percentage decrease of 64% between the average number of whooping signals recorded between midnight and 1am (33/hour), and those recorded between 12 and 1pm (17/hour). It can be seen that the curve is smooth with a sharp decrease in the number of whooping signals after 11am. There is also a much more gradual increase after 2pm with larger steps between 5 and 7 pm.

**Fig 5:**
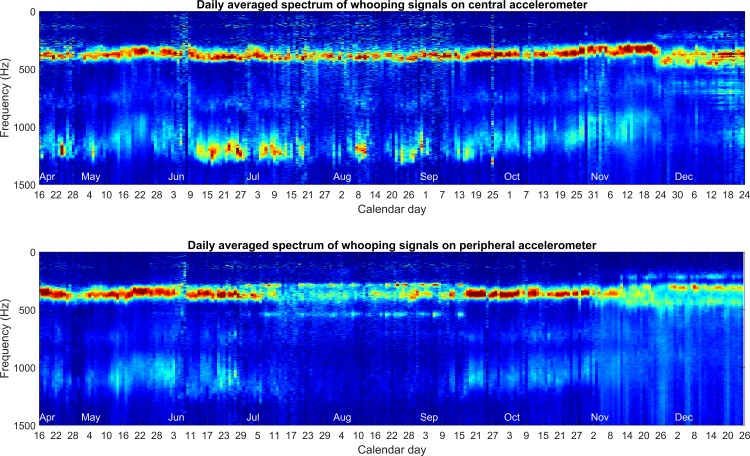
Long-term evolution of the daily averaged spectrum of whooping signals. Data from Apr 18^th^ until Dec 25^th^ 2015 is shown, obtained after the subtraction of the background signal, which would otherwise produce a pronounced peak at 125Hz coming from the bees’ hum.

**Fig 6:**
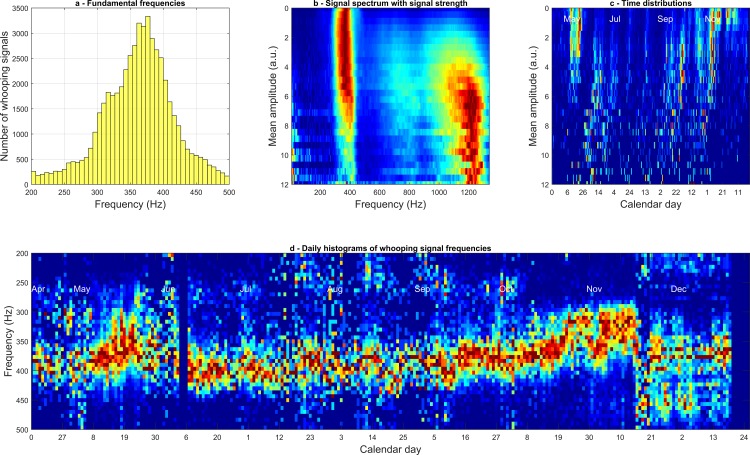
Distribution analysis for data coming from the central accelerometer. **a**—Fundamental frequency distribution; **b**–The averaged spectrum of whooping signals with a specific amplitude displayed in descending order from highest (12 a.u.) to lowest (0 a.u.) amplitude Colour codes the measured amplitude in arbitrary units; **c**–Temporal histograms of whooping signals of a specific amplitude. Colour codes the likelihood of occurrences; **d**—Daily histogram of whooping signal fundamental frequencies.

It can be seen in Fig 5 that the whooping signal spectrum holds very stable at 380 Hz across the whole data set. There is a slight reduction of around 15% in frequency in late May (seen more clearly in Fig 5D and S11B Fig) which is most apparent through examination of the upper harmonics. However, the frequency returns in mid-June until mid-October when the average frequency begins to drop again by around 15%. The amplitude of the third harmonic around 1200 Hz can be very high mostly on the central accelerometer, revealing emitter bees in the close vicinity of the accelerometer (see Fig 6C).

The graphical representation of the statistics for the peripheral accelerometer mirrors that of the central and can be found in S11 Fig.

Fig 6A shows that on the central accelerometer the majority of whooping signals exhibit a fundamental frequency between 300 and 450Hz. Relatively few signals occur at less than 250Hz or above 500Hz. This is confirmed in Fig 6D showing that the majority of signals occurring on a daily basis occur between 280 and 460Hz. There is negligible effect of time/season on peak frequency *(Central*: *R*^*2*^ = *0*.*001*, *p<0*.*001; Peripheral*: *R*^*2*^*(157521) = 0*.*004*, *p<0*.*001)* across either channel (Fig 6D and S11D Fig) providing further evidence that the signal spectrum holds stable throughout the active months. Note that there are slight oscillations apparent in the fundamental frequency of whooping signals, particularly on the central accelerometer during the summer, and this does appear to be correlated with the brood cycle (see S12 Fig). Additionally, there is also a tail-off of around 20% towards the end of May and October that is somewhat apparent in Fig 5. There appears to be a slight positive relationship between signal amplitude and frequency in the centre (Fig 6C) and the periphery (S11C Fig) (Central: *r*_*s*_ = *0*.*1032*, *p <0*.*001;* Peripheral: *rs = 0*.*06*, *N = 157521*, *p <0*.*001*) with the louder signals also exhibiting a more pronounced upper harmonic peak. However, a minority of low frequency signals actually exhibit a stronger second harmonic as seen in S14 Fig. The amplitude shows no daily trend in either the central (Fig 6C) or the peripheral channel (S11C Fig) (Central: *R*^*2*^ = *0*.*04*, *p = 0*.*16; Peripheral*: *R*^*2*^*(157521) = 0*.*043*, *p = 0*.*12*), strongly suggesting that the effect demonstrated in Fig 6C and S11C Fig can take place at any point in time, and is most probably a waveform spectral change due to the distance that the wave has propagated within the honeycomb.

### Supporting video evidence

A head butting event leading to trophallaxis is demonstrated in S2 Movie and S3 Movie of the Supplementary Material, with the sound track coming from an accelerometer embedded in the honeycomb under video analysis. It demonstrates a lag around 200 ms between the head butting and the whooping signal. Two accidental bee to bee collisions are shown in S4 Movie and S5 Movie, with whooping signals elicited without measurable time lag. S6 and S7 Movies are excerpts of recordings from both sides of a frame simultaneously, they demonstrate how often these signals can take place without any matching visual phenomena (32 in the first 30 seconds alone). S8 and S9 Movies provide evidence of bees producing whooping signals with no clear communicative motivation.

An electromagnetic coil with a moving metal core (ZHO-1364S-36A13, ZenHen LTD., China) was secured to the broodbox of our observation hive to allow repeated mechanical knocks to be provided to the colony. The voltage was set to 9 Volt and knocks were provided every second. Whooping signals are elicited en masse, with a distinct similar lag (200 ms) between stimulus and response, and habituation is clearly demonstrated with a substantial reduction of the bee response after the second stimulus, in Video V10.

### Whooping signals and weather

The effect of outside weather parameters on whooping signal occurrences for the UK dataset is provided in S8 Fig, together with its subsequent statistical analysis.

### French dataset

**Fig 7:**
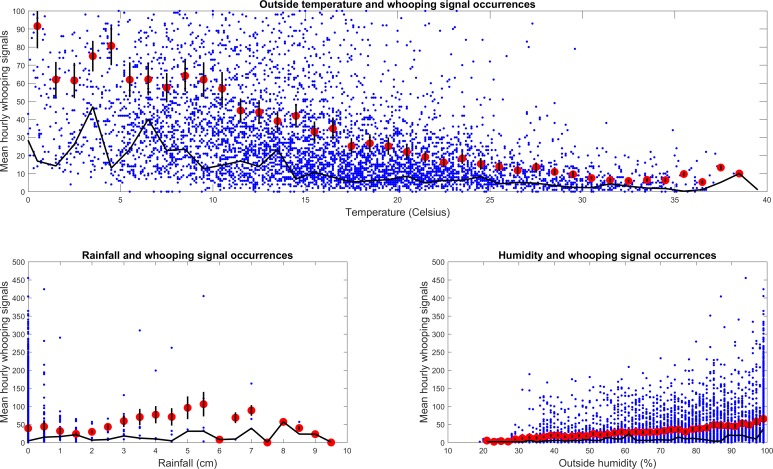
Hourly number of whooping signals with weather. French dataset with corresponding: **a**—average outside temperature; **b**—cumulative rainfall, and **c**—average outside humidity. Red dots indicate the average number of whooping signals with black bars displaying ± 1 SE. The black curve on each graph shows the modal hourly whooping signals.

It can be seen in Fig 7A that there is a negative effect of outside temperature *(B = -0*.*16*, *p <0001)* on whooping signal occurrences with the greatest number of whooping signals being recorded at temperatures around 0. This can be seen by both modal and mean number of whooping signals. There appears (Fig 7C) to be a gradual increase in the mean number of whooping signal occurrences with increased outside humidity *(B = 0*.*286*, *p < 0*.*001)*. However, the modal number of whooping signals does not exhibit a clear trend. There is no overall effect of rainfall on the number of whooping signals *(B = 0*.*032*, *p = 0*.*249)* but it can also be seen that the majority of days saw very little precipitation.

**Fig 8:**
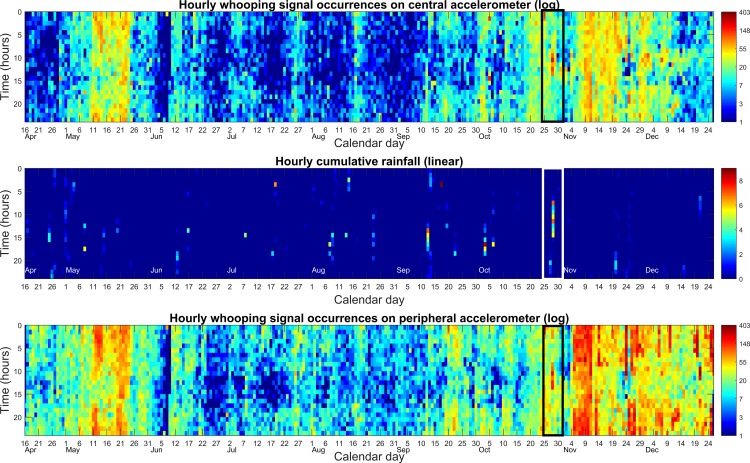
Hourly Whooping signals occurrences with continuous rain. Central (top) and peripheral (bottom) accelerometers. Hourly rain is displayed in the central chart. Highlighted on all three plots is the 28^th^ October 2015.

Fig 8 highlights how rare rainy days have been throughout the duration that the colony has been monitored for. The rectangles draw the attention on the data around the 28^th^ October 2015. This is the only example where it heavily rained throughout most of the day, and it can be seen that there is a reversal in the usual daily trend of whooping signal occurrences, resulting in a lunchtime increase in whooping signal occurrences. A similar phenomenon takes place on the rainy days of the 11^th^ September and 3^rd^ October, where whooping signals appear continuously throughout the day, exhibiting no reduction at lunch time.

**Fig 9:**
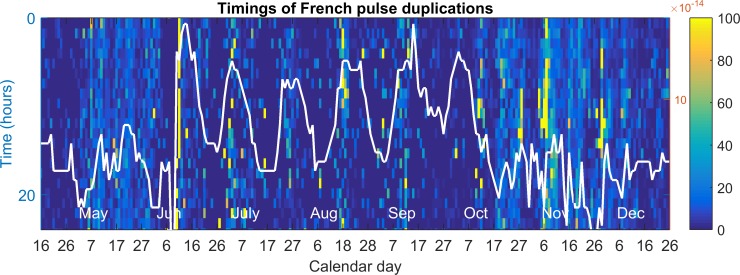
Time course of the percentage of duplicated pulse detections on both channels. Dark blue is 0% duplications; yellow is 100% duplications. The modal daily accelerometer signal amplitude distribution is superimposed over the top, with a white line and acceleration given on the right hand side in mm/s^2^.

### Duplicated pulse detections

It was found that 6.5% of the entire collection of whooping signals were detected on both channels. Fig 9 shows that the highest modal peak amplitude, known to follow the brood cycle in the summer season [28], coincides with the highest occurrences of duplications. Channel-wise analysis of non-duplicated pulses (S13 Fig) revealed that the amplitude of whooping signals detected on the peripheral accelerometer *(median*: *31*.*6 a*.*u; mean*: *38*.*48 a*.*u; SE*: *+/- 0*.*35)* was greater than on the central accelerometer *(median*: *17*.*8 a*.*u; mean*: *22*.*5 a*.*u; SE*: *+/- 0*.*236)*. Wilcoxon’s Signed Rank test confirmed that duplicates had a higher amplitude on the peripheral accelerometer *(Z = 48*.*937*, *p < 0*.*001)*.

## Discussion

### Whooping signal commonness and its implications

Never seen in any previous study, the most striking outcome of our work is the very high level of occurrences that these pulses exhibit within the detection range (approximately 3.5cm) of the accelerometers: up to 10 times per minute over both accelerometers (which monitor approximately 4r^2^π = 153cm^2^, i.e. 1% of the hive’s total honeycomb surface area, 2x10x35x21 cm^2^), that can be extrapolated to a potential of 960 per minute over the whole colony, not counting bees in the super(s). This has strong implications that there is more to this pulse than *exclusively* being a waggle dance inhibitor. Our results show that the majority of signals occur throughout the night, with a distinct decrease by 50% at around midday. During the night, no bee (and certainly no scout bee) ventures outside. Furthermore, it has been previously demonstrated that during the night, foragers enter a sleep-like state [29, 30]. Therefore, it seems most implausible that at this time waggle dances would be taking place. In fact, Schneider [31] measured the time-course of hourly waggle dance occurrences that almost shows the exact opposite trend to that shown in Fig 4 and S4 Fig (However, Schneider’s [31] observations only took place throughout the day time). This signal is also very frequent in the winter months when the colony is over-wintering. Again, it is most unlikely that waggle dances, or even foraging, would be taking place at this time. Furthermore, Seeley *et al*. [27] suggested that this signal plays an integral role in the collective decision making during swarming. However, our figures show homogeneous results in terms of occurrence in the hours/days surrounding the primary and secondary swarms. The lack of signals throughout the day, except on days of heavy rain, also suggests that the majority of honeybee whooping signallers are older bees (i.e. foragers). In addition, we see a lack of overall correlation of hourly occurrences with rain on both datasets, but for this signal to be a waggle dance inhibitor, it is expected that we would see a negative effect of rainfall on whooping signals.

### Stop signal spatial distribution

The majority of trends and statistics are concordant across the two channels, giving further confidence in the results. It suggests that activities associated with whooping signals do not alter based upon the bee’s location on the comb. However, it was seen that around twice more whooping signals were observed at the periphery of the frame in both the French and UK data sets. On the UK data set, it is also seen that there is a return of whooping signals in October on the central accelerometer. It is unclear what caused this drop off in signals but the continuing of the whooping signals being recorded at the centre whilst they disappear from the periphery could be an effect of the bees gathering in a central area forming a “winter cluster" [32]. This phenomenon is not observed for the French dataset and this is likely due to the exceptionally mild winter season experienced in 2015: with temperatures remarkably exceeding 20°C around Christmas, it is unlikely that a winter cluster would have formed.

### Extensive characterisation of whooping signals

In the supplementary results further physical characterisation of honey bee whooping signals (S14 Fig) is provided. We show the average of whooping signals that occur at similar frequencies, thereby removing the broadening of the spectrum that occurs when a daily average is calculated (Fig 5) over whooping signals produced over the full range of possible fundamental frequencies, and showing that whooping signals exhibit very narrow Fourier peaks (coming from a perfect sinusoidal oscillation) and very well defined upper harmonics. The daily averaged spectrum of all whooping signals on the UK data set shows that throughout the more active months, the frequency of the whooping signal is very stable at around 320Hz, and 16% lower than the 380Hz observed on the French dataset. However, both the values are fitting with Lau and Nieh [9] who claimed that stop-signals had an average duration of 0.14s and an average fundamental frequency of 328.3 ± 58.8 Hz. Peak frequency and daily histograms for both data sets show no other obvious trend than stated previously, further supporting the finding that the signal frequency holds stable throughout the year. Through calculation of the daily averaged spectrum, more evidence is provided showing that whooping signals decrease in terms of number and frequency (Hz) from late September until the end of the data set in November. The reduction in occurrences on the UK can be attributed to the ever declining population in the lead up to the colony failure. It is seen on the French data that whooping signals actually increase during the winter, but this may be an artefact of a decreased honeycomb mass density.

The apparent reduction in signal frequencies that are found during the winter months of both datasets, and also after the last swarm of the French data, is highly robust. It appears to be occurring at times when the brood cycle is interrupted and thus the mean age of the worker population is temporarily increasing. It is therefore possible that whooping signal frequency can give an indication of the average overall age of the worker bees.

### Modulation of measured whooping signals by honeycomb status

The frequency of the signal appears to correlate positively with its amplitude and also louder signals exhibit more of the upper harmonics than quieter ones. However, a minority of low frequency whooping signals actually exhibit a stronger second harmonic as seen in S14 Fig. The lack of trend in signal peak amplitudes over time seen over both datasets suggests that the detected amplitude of each pulse is a result of the distance between the accelerometer and the individual signalling bee. The relative amplitudes of Fourier Peaks may therefore provide a means to estimate that distance.

Upon analysis of pulses detected on one channel only, it was found that the signal amplitude was significantly greater on the peripheral accelerometer. At the periphery it has been shown that by detaching the honeycomb from the frame honeybees are able to amplify their signals across areas not restricted by the support [33]. In the centre of the comb the frame load would also usually be much greater, with honey, pollen and brood being stored there [6]. Signals being sent in this region would therefore be more severely attenuated than those sent at the periphery [6]. This is one likely explanation for the difference in the number of whooping signals detected between the two channels on both datasets. Duplicated results also give us an indication of the range of detection that each accelerometer had. The accelerometers were placed 7cm apart with only 6.5% of signals being duplicates. When the honeycomb is fully loaded, no duplications are found, suggesting a radius of sensitivity less than 3.5cm for each accelerometer. However, when the honeycomb is light, the low level of duplications suggests a radius of approximately 3.5cm. The modal amplitude in Fig 9 is fairly stable at the centre throughout April and May. This suggests that the large increase in whooping signal detection seen between April and June is genuine and not the consequence of lower honeycomb mass density.

It is seen throughout our results that the honeycomb mass density does influence the number of detected pulses. From a daily perspective, however, over the course of 24hours the change of the brood is minimal, and so is the change of the sensitivity of the accelerometer. The hourly trend in whooping signal occurrences as seen in Fig 4 is therefore a genuine phenomenon and not an artefact of accelerometer sensitivity. In addition, the brood cycle has finished upon entering the winter months and thus the fluctuations in signal amplitude can be attributed to the overall activity of the bees.

### Whooping signals variations with weather

On the French dataset days of precipitation are actually quite rare, skewing the analysis. This could explain why, overall, rainfall had no effect on whooping signal occurrences. The decrease in whooping signals with temperature within the French data contradicts that of the UK dataset and what we were expecting: an increase in temperature would elicit an increase in activity and thus whooping signals. The days of temperatures below 5°C were uncommon over the period of data collection but the trend is not affected if this data is removed from the analysis, suggesting it is genuine. Humidity appeared to have a slight positive effect on the number of whooping signals in the French colony, however no trend is seen for that of the UK. This contradiction of results between the French and UK colony may suggest that weather can affect colonies differently depending on other intrinsic or extrinsic factors. Daily and seasonal trends are in any case much more robust than trends correlating whooping signals to weather, perhaps due to the excellent ability of the honeybee to thermo-regulate within the colony.

A possibility is that the signal that we have analysed serves as a warming mechanism. This is somewhat suggested by Fig 7, the enhanced occurrences in the winter in Fig 8 and the night time whooping signal maxima. Closer inspection however reveals that the coldest day in the year (Dec 3^rd^) exhibits a decrease in occurrences, and that the high occurrences taking place in May correspond to an exceptionally mild spring (daily temperatures ranging from 15 to 28°C), whilst coldest daily temperatures are known to usually take place at sunrise, where Fig 4 does not exhibit a maximum. Our data, therefore, does not provide support for the idea that the whooping signal is also associated with warming.

The results that we are showing encompass long time durations, during which the colony status will change substantially, which may result in artefacts due to ‘statistical non independence’. If data on numerous colonies simultaneously recorded was available, an interesting future study would be to do such correlation plots at specific times of the year, to somewhat remove these artefacts that presently lie within our data.

### More than just an inhibitory signal

In the videos where whooping signals are seen as a result of a collision between two bees, there is no waggle dance or trophallaxis present on the frame under investigation. A measurable time lag around 200 ms often separates the collision from the vibrational pulse although there are instances without any measurable lag.

Evidence of this features within our work, suggesting that whooping signals increase at times when honeybee density increases within the hive when members of the foraging caste would be considered to be in a state of rest, i.e. at night, in winter and at times of heavy rain. This would lead to a greater potential for surprise collisions to occur within the more crowded, less-sensitised hive.

In the majority of cases numerous instances of whooping signals can be logged without any visual evidence of any specific phenomenon taking place on the honeycomb (see Supplementary S6 Movie). In other instances, all our observations reveal whooping signals as a startle response or linked with trophallaxis, usually with a measurable reaction time around 200ms. Whooping signals elicited en masse as a startle response can sometimes also be demonstrated without any sophisticated equipment, when collecting a swarm into a cardboard box and leaving it undisturbed for approximately 20 minutes. When lifting the box, beekeepers will be familiar with the distinct ‘wooh’ sound emanating from the box, acting as a speaker membrane.

This in combination with statistical trends, leads us to the proposition that this pulse cannot be *exclusively* be limited to the definition of ‘stop signal’ but can be detected under many different circumstances with the addition of being a startle response to an unexpected stimulus. Following this suggestion, signal occurrences may therefore reveal a mixture of bee density, overall colony agitation, and inhibitory or trophallaxis request head-butting events.

Further work would be to investigate this pulse when it is associated with a startle response. It will require accelerometer measurements where we artificially startle bees in the vicinity of the sensor within an observation hive. We will be able to quantitate the extent to which a bee becomes habituated to the startle stimulus. When applied to the entire colony, e.g. at a random time of the day to avoid conditioning, it will be possible to assess the extent to which the response changes on the long term, providing an indicator of the status of the central nervous system of the average worker of a colony, similar to what is presently done in humans and mammals.

To conclude, the results of this study show that this pulse occurs within a variety of contexts, which in turn acts to unify all previous author’s results. In future work, authors should therefore exercise caution in assigning specific interpretations.

## Supporting information

S1 File A description of the process of principal component analysis and discriminant function analysis to discriminate between true and non-whooping signals. This includes text as well as 4 associated figures: Figure A. A comparison of genuine stacked whooping signals spectrograms (top) to that of the falsely detected rain droplets (bottom). Although critical listening allows easy discrimination, visual investigation of the spectrograms reveals high similarities, causing our algorithm to undertake spurious detections, in its “first pass”. Figure B. Outcome of the supervised clustering of whooping signals (red cloud) and rain droplets (blue clouds) for discrimination, shown in DF space. The overlap is negligible and well below 1%. Figure C. Hourly occurrences of queen/worker pipes within the French data set. Colour intensity denotes the hourly number of queen pipes or worker pipes. Figure D. Hourly occurrences of high amplitude spikes within the French data set (caused by bees maintaining the wax in the immediate vicinity of the accelerometer). Colour intensity denotes the hourly number of spikes.
(DOCX)

S1 Fig Pictorial representation of the algorithm (first pass) behind our detection software. Rather than matching spectra, spectrograms are compared, providing a more specific criterion for detection, suitable for the highly repeatable features of the whooping signal.
(DOCX)

S2 Fig Comparison of the three matching strategies for the detection software. The reciprocal of the Euclidean distance (black line) gives similar importance to the spectrogram high and low signal intensity. The cross correlation product (red line) promotes the information found in the high intensities of the spectrogram (peaks). The ratio of the red to black (blue curve) gives the best outcome, when all curves are normalised to make noise levels (low criterion amplitude areas outside the central peak) identical.
(DOCX)

S3 Fig Hourly occurrences of whooping signals on the central and peripheral accelerometers of the UK hive (2014 season). The colour codes the number of occurrences on a linear scale.
(DOCX)

S4 Fig Average number of whooping signal occurrences observed for each hour of the day over the vibrational dataset shown in the previous figure. The vertical bars indicate +/-1 SE.
(DOCX)

S5 Fig Long-term evolution of the daily averaged spectrum of whooping signals from Jul 18^th^ until Nov 9^th^ 2014 of the UK hive dataset. Data was obtained after the removal of the background signal, which would otherwise produce a pronounced peak at 125Hz coming from the bees ‘buzzing’.
(DOCX)

S6 Fig Statistics of whooping signals from the central accelerometer within the UK hive. S6a: histogram of all whooping signal’s fundamental frequencies recorded on the peripheral accelerometer; S6b: Averaged spectrum of whooping signals for a specific amplitude; S6c: The evolution of whooping signal amplitude histogram over time; S6d: Daily histogram of whooping signal fundamental frequencies.
(DOCX)

S7 Fig Statistics of whooping signals from the peripheral accelerometer within the UK hive. S7a: histogram of all whooping signal’s fundamental frequencies recorded on the peripheral accelerometer; S7b: Averaged spectrum of whooping signals for a specific amplitude; S7c: The evolution of whooping signal amplitude histogram over time; S7d: Daily histogram of whooping signal fundamental frequencies.
(DOCX)

S8 Fig hourly number of whooping signals with corresponding: a (top left): average outside temperature; b (top right): average outside humidity; c (bottom left): cumulative rainfall, and d (bottom right): average atmospheric pressure. Red dots indicate the average number of whooping signals with black bars displaying ± 1 SE.
(DOCX)

S9 Fig Matlab® extraction of Michelsen’s begging signal published in 1986. **a**- The original waveform from the publication. **b**- The waveform extracted with MATLAB®. **c-** The time differential of the previously extracted waveform, providing acceleration as a function of time.
(DOCX)

S10 Fig Spectrogram of Michelsen’s [21] begging signal, after it has been processed as shown in S9 Fig. (DOCX)

S11 Fig Distribution analysis for data coming from the peripheral accelerometer. **a**—Fundamental frequency distribution; **b**–The averaged spectrum of whooping signals with a specific amplitude displayed in descending order from highest (12 a.u.) to lowest (0 a.u.) amplitude Colour codes the measured amplitude in arbitrary units; **c**–Temporal histograms of whooping signals of a specific amplitude. Colour codes the likelihood of occurrences; **d** -Daily histogram of whooping signal fundamental frequencies.
(DOCX)

S12 Fig The daily histograms of whooping signal fundamental frequencies derived from the centre (a) and periphery (b) of the honeycomb. As in Fig 9, the modal daily accelerometer signal amplitude distribution is superimposed with a red line and acceleration axis given on the right hand side in mm/s^2^.
(DOCX)

S13 Fig Comparison of the peak amplitude of all whooping signals recorded on the central and peripheral accelerometers. The red line denotes the median, x is the mean, and indents show the confidence intervals at 95%.
(DOCX)

S14 Fig Extensive characterisation of honeybee whooping signals on the French dataset. Whooping signals spectra of similar fundamental frequencies were averaged and stacked from left to right. The amplitude is displayed in a logarithmic scale with yellow denoting high amplitude and dark blue denoting low amplitude.
(DOCX)

S15 Fig Image of the electromagnetic coil secured laterally to the brood box of the observation hive used in S10 Movie. (DOCX)

S1 Movie This video displays the waveforms (blue/ orange for left/ right channel) that can also be heard in the audio. The corresponding spectrograms of a random selection of 245 whooping signals detected by our software are also shown (bottom).
(WMV)

S2 Movie The video shows one frame of our 2016 observation hive, with two accelerometers embedded in the honeycomb. The accelerometer closest to the event of interest (the left hand side one) output was fed into the video recorder, providing the sound track of the footage. The coaxial cable of the accelerometer can be seen. The window highlighted with a white rectangle is scaled up on the right of the image, below the spectrogram of the accelerometer track. A collision is clearly seen, followed by a whooping signal, followed by trophallaxis.
(AVI)

S3 Movie The same video as S2 Movie is provided, with a frame rate decreased from 50 to 15 frames per second. (AVI)

S4 Movie The same set up as for S1 Movie, showing an accidental collision between a falling bee and another one on the frame, generating a whooping signal that is synchronous with the event. Note that a louder whooping signal can be heard immediately before the collision, coming from an event not identified on the frame under investigation.
(WMV)

S5 Movie The video shows one frame of a different observation hive, in 2015, with a single tri-axial accelerometer embedded in the centre of the honeycomb. The accelerometer x and y outputs were fed into the video recorder, providing the stereo sound track of the footage. The protruding accelerometer can be seen. The spectrograms of each accelerometer x and y output are shown. An accidental collision is clearly seen, in near perfect synchrony with a whooping signal.
(AVI)

S6 Movie The video (with the same set up as S1 Movie) shows one side of the frame (we film both sides simultaneously) and is a demonstration of how often these whooping signals can naturally occur within the vicinity of the accelerometers without any corresponding visually evident bee activity/interaction. Throughout the first 30 seconds alone, 32 whooping signals are recorded without any corresponding visually matching evidence or phenomena that we can see. The audio is stereo with the left side accelerometer corresponding to left channel and the right side accelerometer to the right channel.
(AVI)

S7 Movie The video is a simultaneous recording of the other side of the frame in S6 Movie. The audio has been inverted to match the channel to the corresponding side.
(AVI)

S8 Movie The video (with the same set up as S1 Movie) shows a bee closing its wings in perfect synchrony with the detection of a whooping signal. No direct communication with another bee can be seen.
(WMV)

S9 Movie The video (with the same set up as S1 Movie) shows a bee producing two successive rapid wing movements in perfect synchrony with detected whooping signal vibrations. No direct communication with another bee can be seen.
(WMV)

S10 Movie The video shows our set up with our 2015 observation hive. The spectrograms of each uniaxial accelerometer output are shown. The broodbox is stimulated with an electromagnetic coil with a moving inner metal part, secured to the side of the broodbox as shown in S15 Fig, with a 9 Volts pulse, once every second. The whooping signals elicited en masse are clearly heard and seen on the spectrograms, with decreasing response as the stimulus is repeated, and a distinct time lag around 200 ms between stimulus and honeybee response.
(AVI)

S1 Audio This is an hour long, accelerometer recording from the heart of our honeybee hive in Jarnioux, France. The track was recorded on the 12^th^ May 2015 between midnight and 1am, and is a whooping signal hotspot as identified by Fig 3. This particular track was chosen because it allows the reader, by critical listening, to (1) appreciate how often this signal occurs naturally, (2) further demonstrate that our hotspots are genuine, and (3) hear how different whooping signals are compared to the other various signals that can be heard on the track,—including queen toots.
(MP3)

S2 Audio Some of the worker pipes identified and displayed in S5 Fig. Some of the detected timings were used to extract a short excerpt of recording, and these were concatenated one after the other to produce this file.
(MP3)
