# Correction: Long-term trends in the honeybee 'whooping signal' revealed by automated detection

**DOI:** 10.1371/journal.pone.0181736

**Published:** 2017-07-13

**Authors:** Michael Ramsey, Martin Bencsik, Michael I. Newton

The Data Availability statement for this paper is incorrect. The correct statement is:

Data are available from Figshare:

Folder 15: 10.6084/m9.figshare.4815157<https://doi.org/10.6084/m9.figshare.4815157>

Folder 14: 10.6084/m9.figshare.4793653<https://doi.org/10.6084/m9.figshare.4793653>

Folder 13: 10.6084/m9.figshare.4789822<https://doi.org/10.6084/m9.figshare.4789822>

Folder 12: 10.6084/m9.figshare.4772362<https://doi.org/10.6084/m9.figshare.4772362>

Folder 11: 10.6084/m9.figshare.4765705<https://doi.org/10.6084/m9.figshare.4765705>

Folder 10: 10.6084/m9.figshare.4758511<https://doi.org/10.6084/m9.figshare.4758511>

UK Weather Data: 10.6084/m9.figshare.4986725

French Whooping Signals: 10.6084/m9.figshare.4986734

UK Whooping Signals: 10.6084/m9.figshare.4986737
