# Supplementary material for: Long-term trends in the honeybee ‘whooping signal’ revealed by automated detection
Source: PLoS One. 2017 Feb 8;12(2):e0171162. doi: 10.1371/journal.pone.0171162 (PMC5298260; doi:10.1371/journal.pone.0171162)
Supplement: S1 File — This includes text as well as 4 associated figures: Figure A. A comparison of genuine stacked whooping signals spectrograms (top) to that of the falsely detected rain droplets (bottom). Although critical listening allows easy discrimination, visual investigation of the spectrograms reveals high similarities, causing our algorithm to undertake spurious detections, in its “first pass”. Figure B. Outcome of the supervised clustering of whooping signals (red cloud) and rain droplets (blue clouds) for discrimination, shown in DF space. The overlap is negligible and well below 1%. Figure C. Hourly occurrences of queen/worker pipes within the French data set. Colour intensity denotes the hourly number of queen pipes or worker pipes. Figure D. Hourly occurrences of high amplitude spikes within the French data set (caused by bees maintaining the wax in the immediate vicinity of the accelerometer). Colour intensity denotes the hourly number of spikes. (DOCX) [file pone.0171162.s001.docx]

# Principal Component Analysis and Discriminant Function Analysis to Discriminate between True and Non Whooping signals

Data was extensively examined by critical listening to ensure that the software was displaying true whooping signals. Once a histogram of hourly whooping signal occurrences had been generated, hotspots and potential anomalies were identified for further examination in both the UK and French data sets. The calendar timings of these were converted into epoch time (or UNIX time) and a separate software was used to upload the signals at that specific time from within the data set. The signal excerpts were then stacked in an audio file allowing a 1 second gap in between detected pulses, to allow enough time for critical listening.

Any signal or artefact that were found to be falsely detected as a whooping signal were fed into the PCA/DFA software to train the computer to discriminate between them (for further information see Bencsik *et al.* [1]). For example, in some days throughout October and November of the UK data set there were up to 1000 whooping signals detected in one day. The signals from the times of exceedingly high detections were concatenated and analysed by ear. In the UK data this turned out to be a collection of water drop vibrations originating from heavy rain. This was confirmed through analysis of the precipitation data from an onsite weather station. The high level of similarity between the spectrograms of a honeybee whooping signal and that of the vibrational pulse caused by a drop of rain falling onto the hive is demonstrated in Figure A.

*
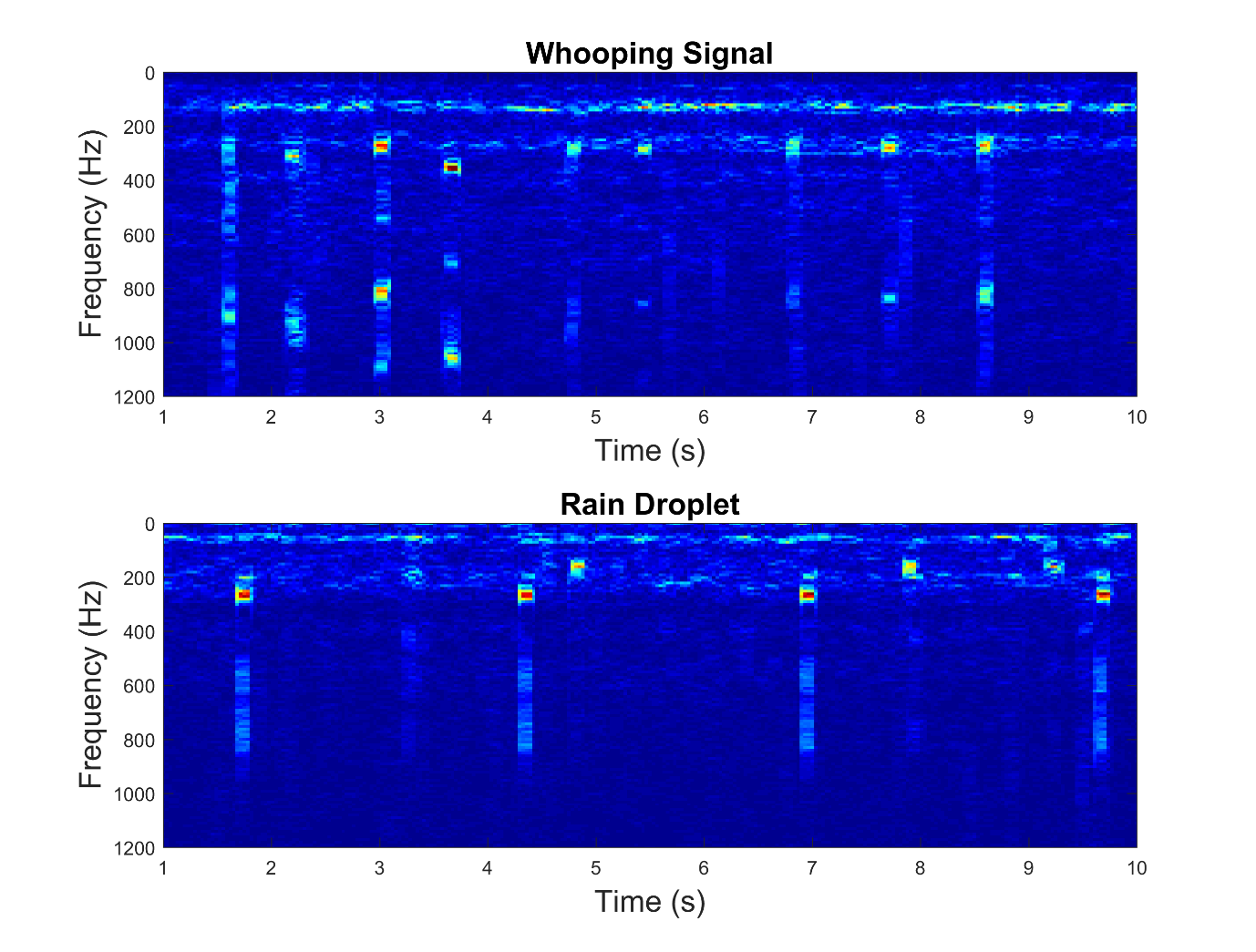
*

**Figure A. Comparison of genuine stacked whooping signals spectrograms (top) to that of the falsely detected rain droplets (bottom).** Although critical listening allows easy discrimination, visual investigation of the spectrograms reveals high similarities, causing our algorithm to undertake spurious detections, in its “first pass”.

Using the DFA algorithm implemented in the Matlab® core, code was developed that runs a second step discrimination analysis. A collection of over 300 droplet timings were selected for comparison to a collection of over 100 true whooping signals that were taken from across the whole data set. These were used to build a ‘training database’ for discrimination by supervised clustering. Each pulse was uploaded into the software allowing 0.5s either side of the recorded pulse time. A spectrogram of the pulse was generated and then centred to allow careful phasing of all detected pulses. The frequencies were then cropped to between 65 and 3000Hz. The spectrogram was coarsened to every other pixel along the frequency axis, in order to both make the spectrum less specific to a particular fundamental frequency value and improve the performance of the software. The pulse amplitude was then normalised by dividing it by its maximum, and finally saved in the training database and this was repeated for all other selected pulses.

The whooping signals and rain droplets were carefully labelled within the training database and their PCA scores calculated. By using a pair of cross correlation products with two discriminant functions identified by the DFA algorithm, the discriminant function coordinates, or ‘DF scores’, could then be calculated. The number of PCA scores (i.e. features of the signals) used in the discrimination was explored until the best discrimination was achieved, identified through the calculation of the percentage error based on how many points from each cluster overlapped. The upper and lower limit of the signal bandwidth were automatically changed and the percentage error was further recorded for optimisation. The parameters that yielded the lowest error in discrimination was used. The coordinates of the centroids of the clusters were calculated and software was developed to provide a threshold for determining whether a whooping signal was genuine or not though calculating its distance to the centroid of each cluster.


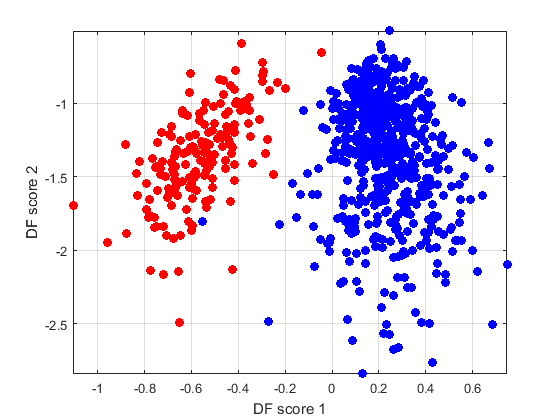


**Figure B. Outcome of the supervised clustering of whooping signals (red cloud) and rain droplets (blue clouds) for discrimination, shown in DF space.** The overlap is negligible and well below 1 %.

The entire data set of whooping signal timings was then put through this procedure. Each pulse was uploaded in turn, its spectrogram was formed, centred, cropped, normalised and coarsened along the frequency axis. Next, the DF coordinates of the detected pulse were calculated by using the pair of cross correlation products and then its distance from each centroid was calculated. The ratio of the two distances was tested to determine which cluster it belonged to. The pulses were then saved as either rain or true whooping signals.

This form of discrimination was also carried out on the French data set. It revealed, in the “first pass” of the detection process that queen pipes, worker pipes and high amplitude spikes had been wrongly detected as whooping signals owing to their similarity in frequency. The same method of supervised clustering was deployed and it allowed us to create separate data sets of queen pipes, worker pipes, and spike occurrences (Figures C and D).

The fact that only whooping signals remain after discrimination was checked by critical listening to hundreds of signals taking place at random times of the year. Specific one-hour long sections of data where hot spots of occurrences were detected were also listened to, and indeed revealed highly frequent whooping signals, as demonstrated in S1 Audio. A movie of stacked whooping signals extracted by our software, with the corresponding audio is also supplied and also further validates the discrimination exercise.

*
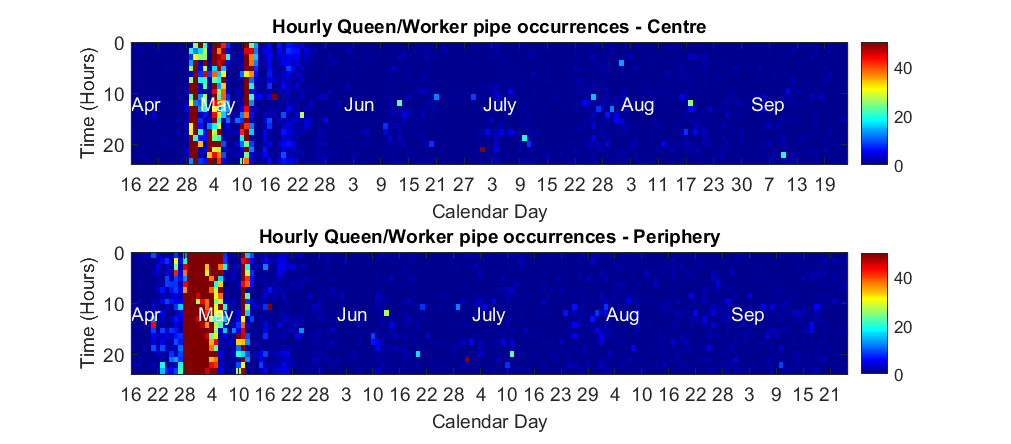
*

**Figure C. Hourly occurrences of queen/worker pipes within the French data set.** Colour intensity denotes the hourly number of queen pipes or worker pipes.

*
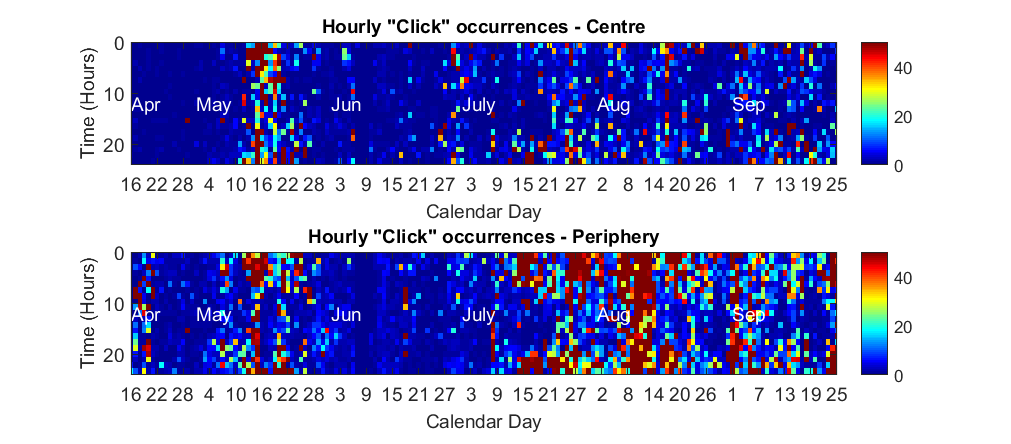
*

**Figure D. Hourly occurrences of high amplitude spikes within the French data set** (caused by bees maintaining the wax in the immediate vicinity of the accelerometer). Colour intensity denotes the hourly number of spikes.

Figure D shows the occurrences of high amplitude spikes generated by bees undertaking maintenance of the wax around the accelerometer.

## References

1. Bencsik, M., Bencsik, J., Baxter, M. and Millet, M. Identification of the honey bee swarming process by analysing the time course of hive vibrations*. Comput. Electron. Agric.* **76**(1) 44-50 (2011)
