# Supplementary material for: Long-term trends in the honeybee ‘whooping signal’ revealed by automated detection
Source: PLoS One. 2017 Feb 8;12(2):e0171162. doi: 10.1371/journal.pone.0171162 (PMC5298260; doi:10.1371/journal.pone.0171162)
Supplement: S1 Fig — Rather than matching spectra, spectrograms are compared, providing a more specific criterion for detection, suitable for the highly repeatable features of the whooping signal. (DOCX) [file pone.0171162.s002.docx]

*
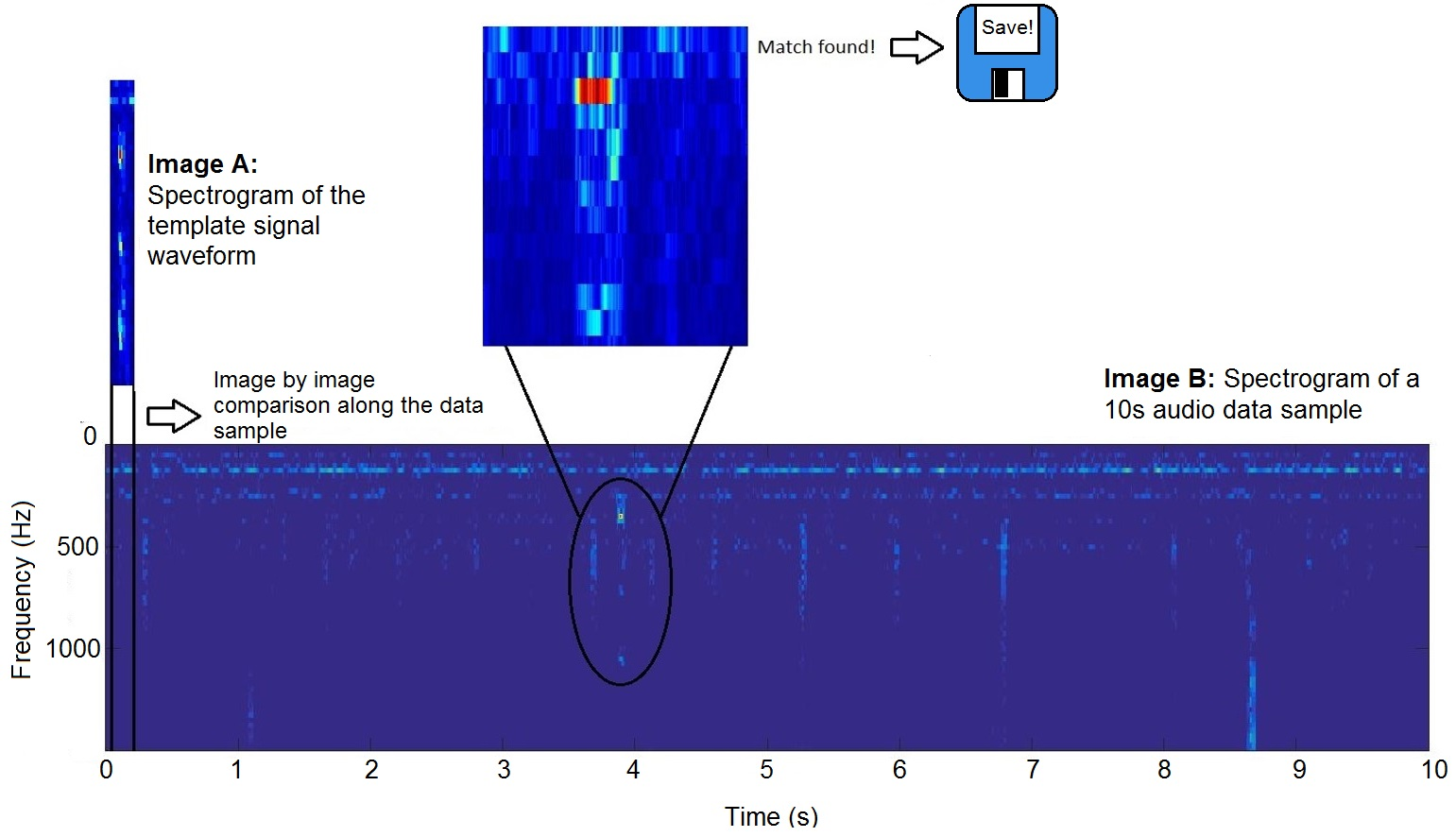
*

**S1 Fig. Pictorial representation of the algorithm (first pass) behind our detection software.** Rather than matching spectra, spectrograms are compared, providing a more specific criterion for detection, suitable for the highly repeatable features of the whooping signal.
