# Supplementary material for: Long-term trends in the honeybee ‘whooping signal’ revealed by automated detection
Source: PLoS One. 2017 Feb 8;12(2):e0171162. doi: 10.1371/journal.pone.0171162 (PMC5298260; doi:10.1371/journal.pone.0171162)
Supplement: S2 Fig — The reciprocal of the Euclidean distance (black line) gives similar importance to the spectrogram high and low signal intensity. The cross correlation product (red line) promotes the information found in the high intensities of the spectrogram (peaks). The ratio of the red to black (blue curve) gives the best outcome, when all curves are normalised to make noise levels (low criterion amplitude areas outside the central peak) identical. (DOCX) [file pone.0171162.s003.docx]

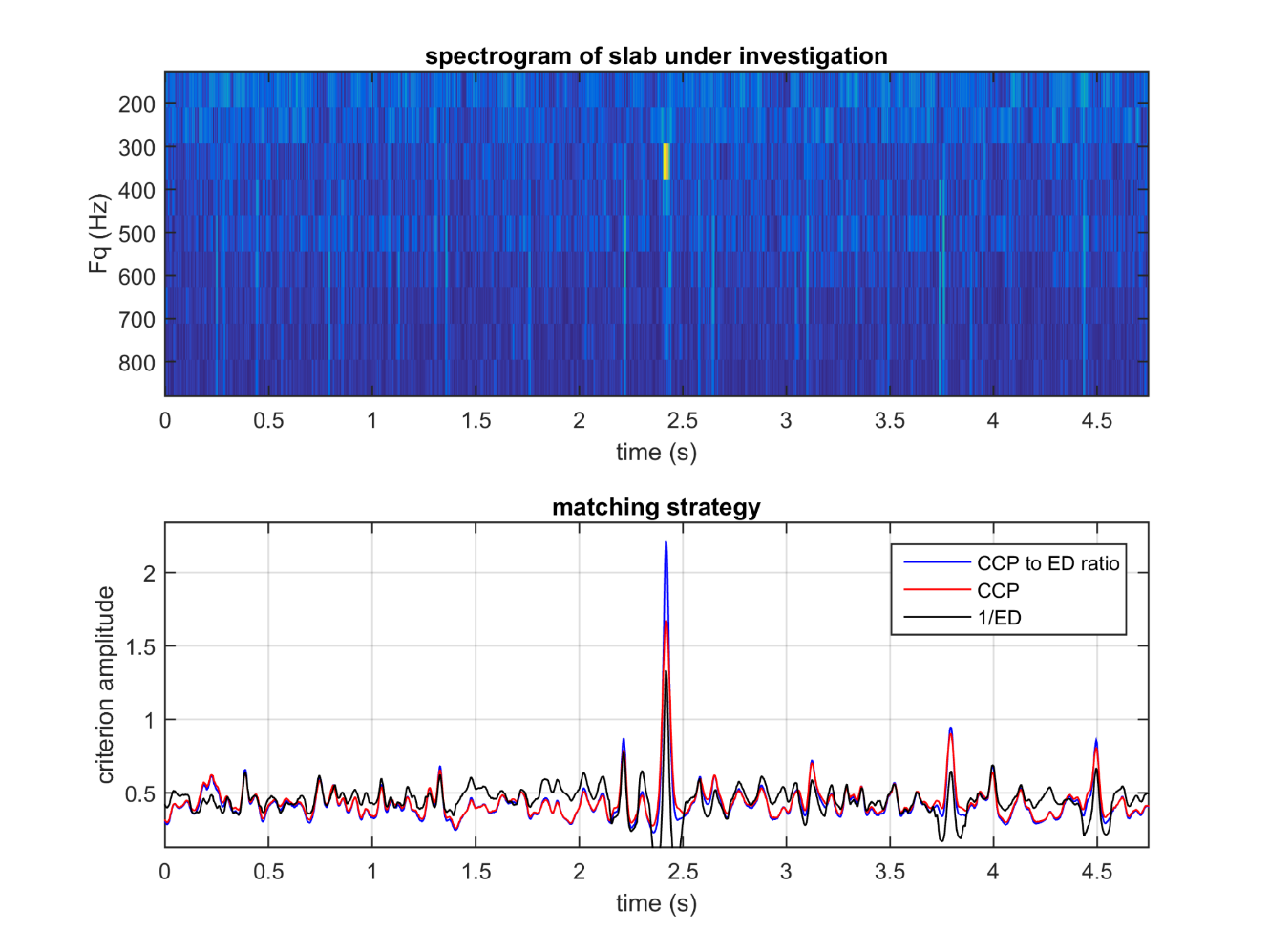


**S2 Fig. Comparison of the three matching strategies for the detection software.** The reciprocal of the Euclidean distance (black line) gives similar importance to the spectrogram high and low signal intensity. The cross correlation product (red line) promotes the information found in the high intensities of the spectrogram (peaks). The ratio of the red to black (blue curve) gives the best outcome, when all curves are normalised to make noise levels (low criterion amplitude areas outside the central peak) identical.
