# Supplementary material for: Long-term trends in the honeybee ‘whooping signal’ revealed by automated detection
Source: PLoS One. 2017 Feb 8;12(2):e0171162. doi: 10.1371/journal.pone.0171162 (PMC5298260; doi:10.1371/journal.pone.0171162)
Supplement: S3 Fig — The colour codes the number of occurrences on a linear scale. (DOCX) [file pone.0171162.s004.docx]

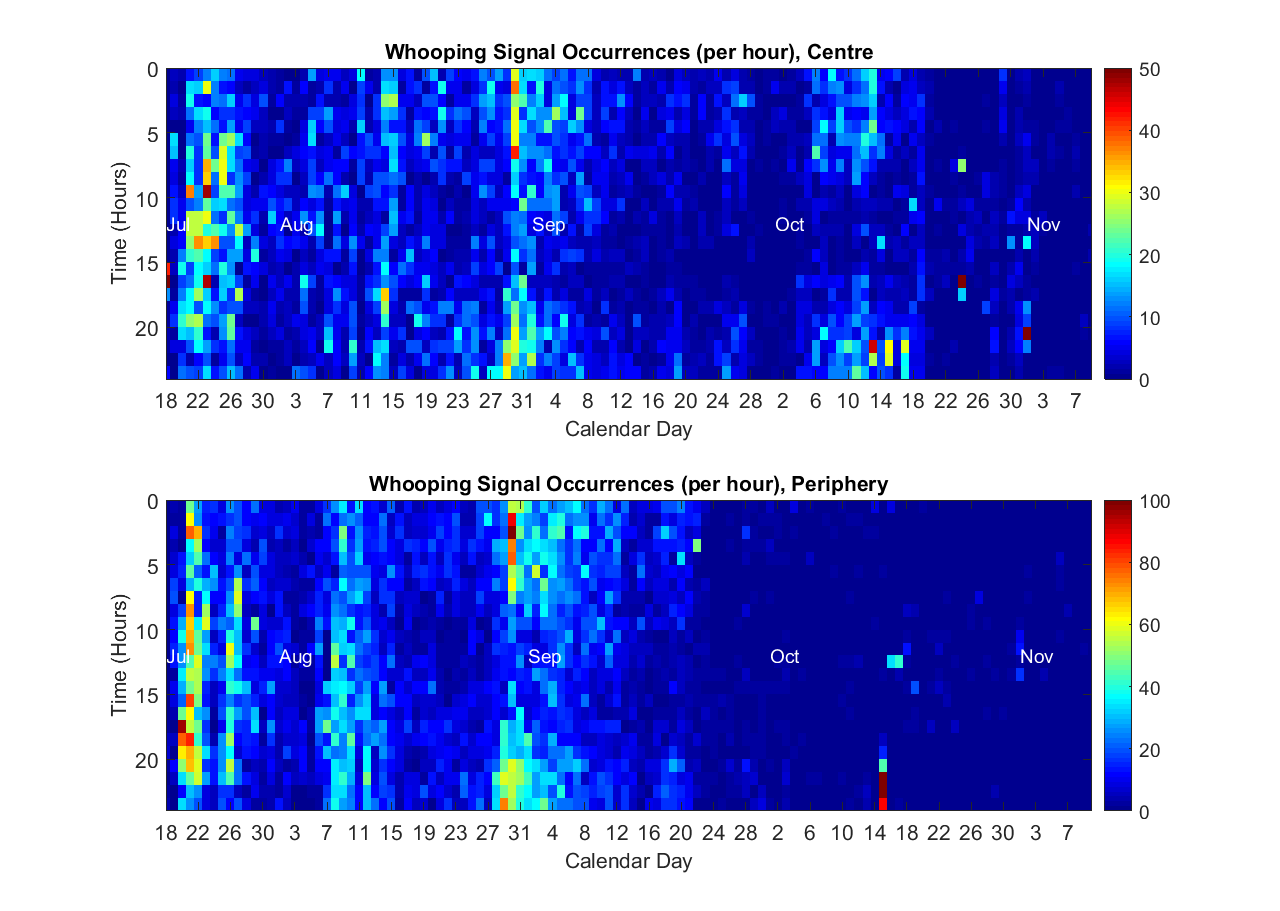


**S3 Fig. Hourly occurrences of whooping signals on the central and peripheral accelerometers of the UK hive (2014 season).** The colour codes the number of occurrences on a linear scale.

S3 Fig illustrates the hourly number of whooping signals that were recorded throughout the 4 months of recording. Whilst slightly less than logged on the French dataset, it still can be seen that whooping signals occur very frequently within the vicinity of the accelerometers (up to 4 times per minute). Similar to the French dataset, there appears roughly 50% less whooping signals detected on the central accelerometer than on the peripheral accelerometer with detections appearing to be modulated by the brood cycle with peaks every 21 - 24 days (see Bencsik *et al.*^31^).

There is also an apparent reduction by around 55% in the number of signals occurring between 11am and 4pm being made more apparent in S4 Fig, where the hourly whooping signals have been averaged over all recorded days. However, this daily trend is not seen as apparently for whooping signals recorded at the end of July, perhaps due to the rainy summer experienced in 2014. The total number of whooping signal occurrences tails off towards the end of September on both accelerometers but returns at the centre towards the end of October, suggesting that this colony did not do well as it progressed into the wintering.
