# Supplementary material for: Long-term trends in the honeybee ‘whooping signal’ revealed by automated detection
Source: PLoS One. 2017 Feb 8;12(2):e0171162. doi: 10.1371/journal.pone.0171162 (PMC5298260; doi:10.1371/journal.pone.0171162)
Supplement: S4 Fig — The vertical bars indicate +/-1 SE. (DOCX) [file pone.0171162.s005.docx]

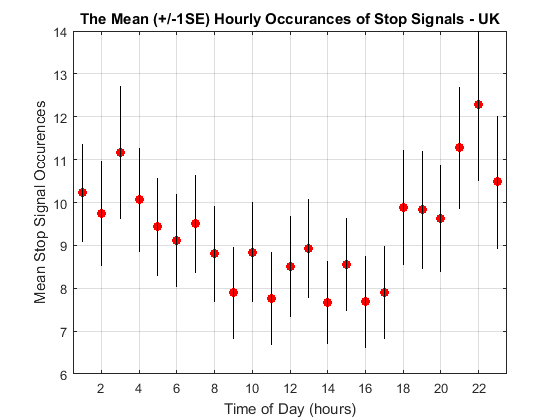


**The Mean (+/- 1SE) Hourly Occurrences of Whooping Signals - UK**

**S4 Fig. The average number of whooping signal occurrences observed for each hour of the day over the vibrational dataset shown in the previous figure**. The vertical bars indicate +/-1 SE.
