# Supplementary material for: Long-term trends in the honeybee ‘whooping signal’ revealed by automated detection
Source: PLoS One. 2017 Feb 8;12(2):e0171162. doi: 10.1371/journal.pone.0171162 (PMC5298260; doi:10.1371/journal.pone.0171162)
Supplement: S5 Fig — Data was obtained after the removal of the background signal, which would otherwise produce a pronounced peak at 125Hz coming from the bees ‘buzzing’. (DOCX) [file pone.0171162.s006.docx]

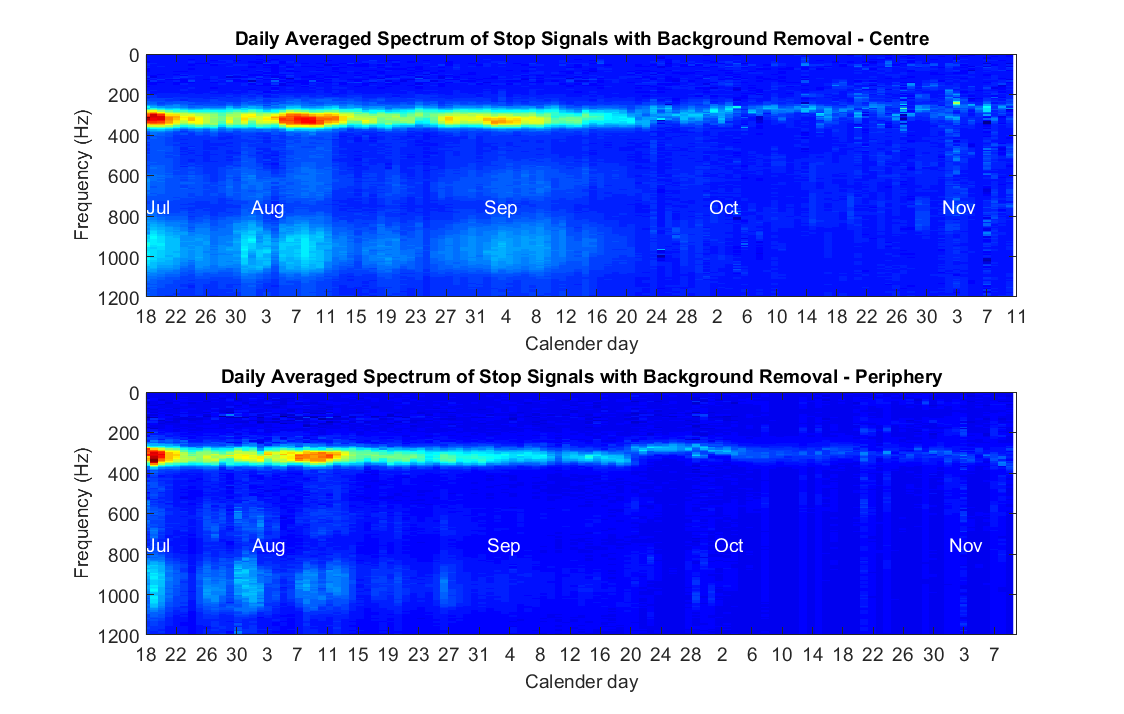


**S5 Fig. Long-term evolution of the daily averaged spectrum of whooping signals from Jul 18^th^ until Nov 9^th^ 2014 of the UK hive dataset**. Data was obtained after the removal of the background signal, which would otherwise produce a pronounced peak at 125Hz coming from the bees ‘buzzing’.

S5 Fig, after removal of the background 125Hz ‘wing buzz’ and its 250Hz harmonic, is showing the long term evolution of the daily averaged spectrum for every whooping signal detected by the software. Unlike the daily average of French whooping signals, it is seen that the signal’s average frequency from the UK hive holds very stable at 320Hz with clouds representing the upper harmonics, throughout the summer months until the end of September where it gradually decreases until the end of the recording in November. Peaks in occurrences take place every 21 days as seen on the French dataset. The average whooping signal frequency taken from all whooping signals in the UK data set was found to be 320 (± 30) Hz, approximately 17% lower than then 380Hz average from the French data set.
