# Supplementary material for: Long-term trends in the honeybee ‘whooping signal’ revealed by automated detection
Source: PLoS One. 2017 Feb 8;12(2):e0171162. doi: 10.1371/journal.pone.0171162 (PMC5298260; doi:10.1371/journal.pone.0171162)
Supplement: S6 Fig — S6a: histogram of all whooping signal’s fundamental frequencies recorded on the peripheral accelerometer; S6b: Averaged spectrum of whooping signals for a specific amplitude; S6c: The evolution of whooping signal amplitude histogram over time; S6d: Daily histogram of whooping signal fundamental frequencies. (DOCX) [file pone.0171162.s007.docx]

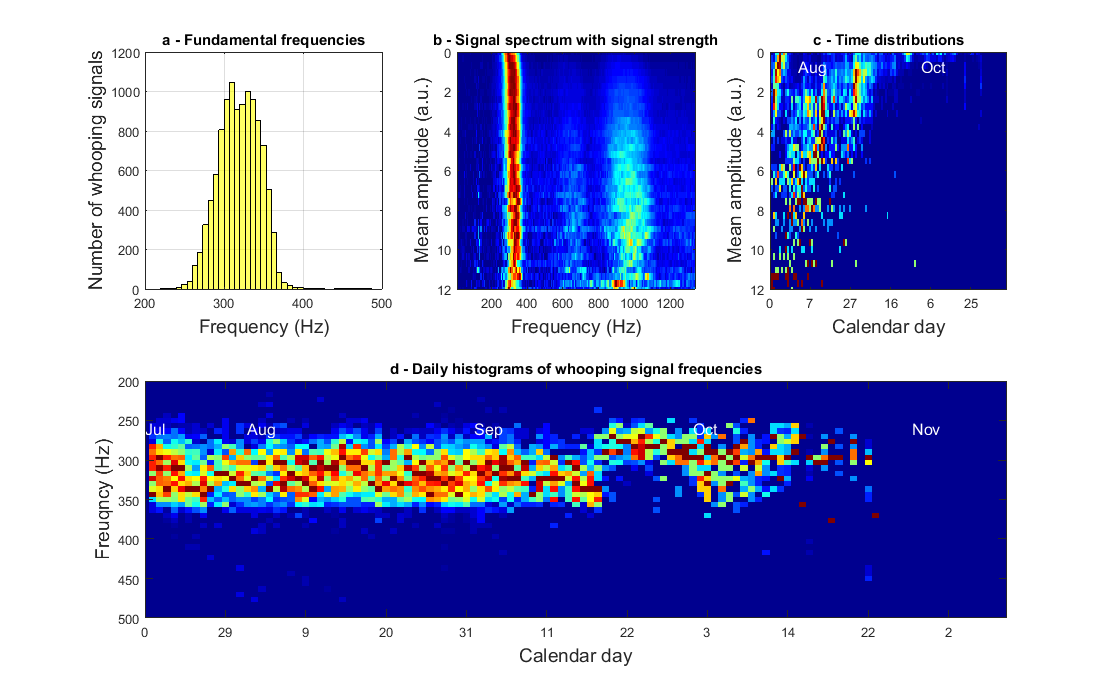


**S6 Fig. Statistics of whooping signals from the peripheral accelerometer within the UK hive.** S6a: histogram of all whooping signal’s fundamental frequencies recorded on the peripheral accelerometer; S6b: Averaged spectrum of whooping signals for a specific amplitude; S6c: The evolution of whooping signal amplitude histogram over time; S6d: Daily histogram of whooping signal fundamental frequencies.

S6a Fig shows that on the central accelerometer, the distribution of fundamental frequencies is approximately Gaussian with the majority of signals occurring between 250 and 370Hz. Relatively few (less than 5%) signals occur at less than 250Hz, or above 380Hz as seen in the French dataset. This is confirmed in S6b Fig showing that the majority of signals occurring on a daily basis occur between 280 and 360Hz. As seen in the analysis of French statistics, there is no effect of time/season on peak frequency *(Ch1: R^2^=0.0000222, p<0.001; Ch2: R^2^= 0.00217, p<0.001)* across either channel (Figs S6c and S7c) providing further evidence that it holds stable throughout the active months. However, a pronounced drop of the frequency towards the end of September / early October is present that is also apparent in S6d Fig. S6d Fig also shows evidence of the return of whooping signals in late October on the central accelerometer that is also apparent in S7d Fig. As for the French data, there appears to be a slight positive relationship between signal amplitude and frequency in the centre (S6b Fig) and the periphery (S7b Fig) (Ch1: *r_(33103)_ = 0.1404, p <0.001;* Ch2: *r_(22202)_ = 0.1775, p <0.001*) with the louder signals exhibiting more of the upper harmonic frequencies and the amplitude shows no daily trend in either the centre (S6c Fig) or the periphery (S7c Fig) (Ch1: *R^2^=0.0002, p=0.16; Ch2: R^2^=0.0033, p=0.12*).
