# Supplementary material for: Long-term trends in the honeybee ‘whooping signal’ revealed by automated detection
Source: PLoS One. 2017 Feb 8;12(2):e0171162. doi: 10.1371/journal.pone.0171162 (PMC5298260; doi:10.1371/journal.pone.0171162)
Supplement: S8 Fig — Red dots indicate the average number of whooping signals with black bars displaying ± 1 SE. (DOCX) [file pone.0171162.s009.docx]

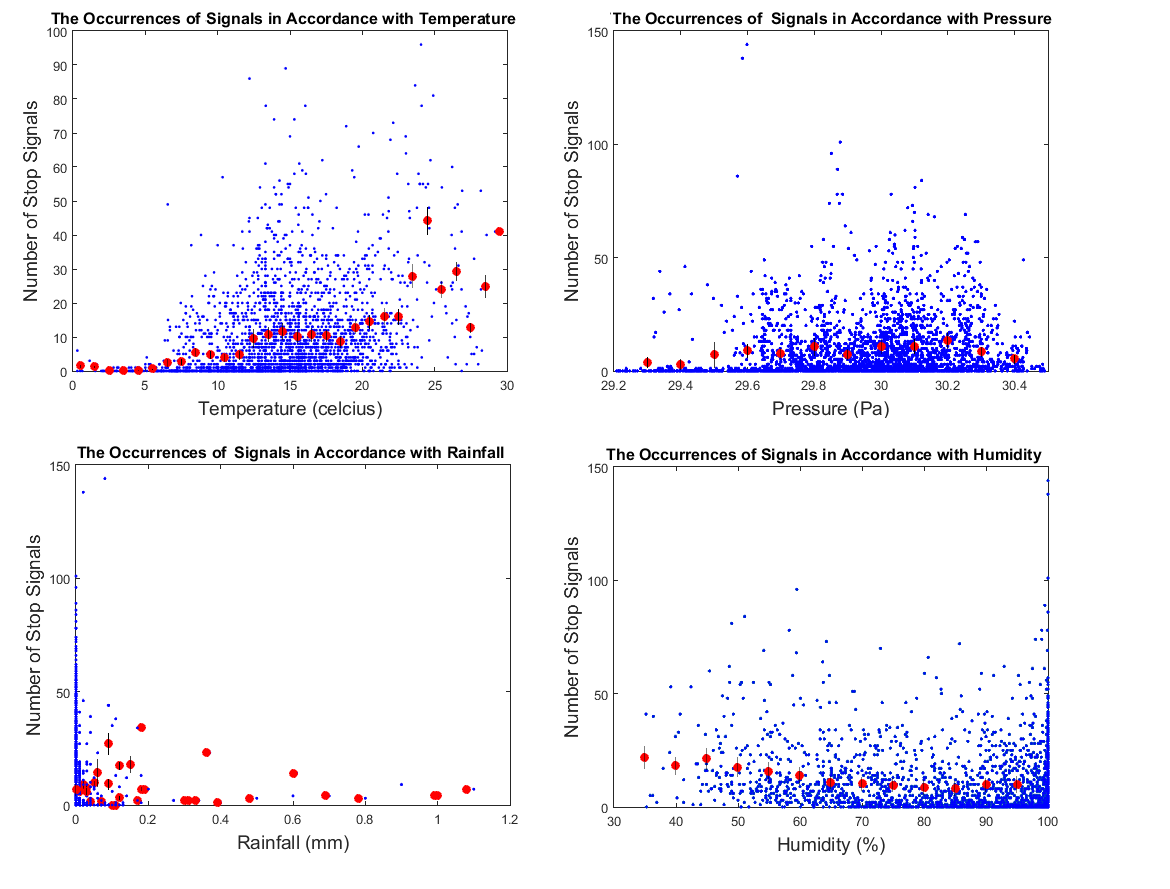


*S8 Fig: hourly number of whooping signals with corresponding: a (top left): average outside temperature; b (top right): average outside humidity; c (bottom left): cumulative rainfall, and d (bottom right): average atmospheric pressure. Red dots indicate the average number of whooping signals with black bars displaying ± 1 SE.*

In spite of substantial scatter, it can be seen in S8a Fig that there is a positive trend of whooping signals with outside temperatures with more whooping signals being recorded when the outside temperature is higher, however there is great variance within the data (*R^2^ = 0.104, t = 13.285, p < 0.001*). This directly contradicts that of the French temperature analysis. The majority of signals occurred between 7 and 23 degrees Celsius, whereas in France the temperature went much higher. There is no significant effect of outside humidity (*R^2^ = 0.042, t = 1.561, p = 0.119*) also contradicting the result of the French analysis. On the other hand, rainfall (*R^2^ = 0.001, t = 1.561, p = 0.853*), and atmospheric pressure (*R^2^ = 0.0014, t = 1.309, p = 0.132*), had no effect on the occurrence of whooping signals. It must be highlighted that the contradictions between the French and UK datasets concern data that is substantially scattered.
