# Supplementary material for: Long-term trends in the honeybee ‘whooping signal’ revealed by automated detection
Source: PLoS One. 2017 Feb 8;12(2):e0171162. doi: 10.1371/journal.pone.0171162 (PMC5298260; doi:10.1371/journal.pone.0171162)
Supplement: S9 Fig — a- The original waveform from the publication. b- The waveform extracted with MATLAB®. c- The time differential of the previously extracted waveform, providing acceleration as a function of time. (DOCX) [file pone.0171162.s010.docx]

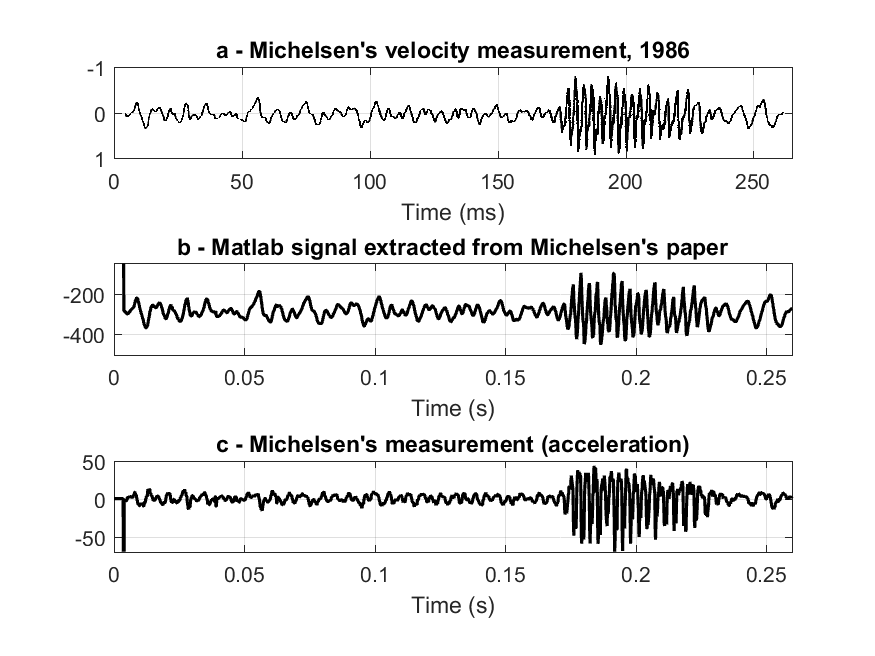


**S9. Fig.** **Matlab® extraction of Michelsen’s begging signal published in 1986.** **a**- The original waveform from the publication. **b**- The waveform extracted with MATLAB®. **c-** The time differential of the previously extracted waveform, providing acceleration as a function of time.
