# Supplementary material for: Long-term trends in the honeybee ‘whooping signal’ revealed by automated detection
Source: PLoS One. 2017 Feb 8;12(2):e0171162. doi: 10.1371/journal.pone.0171162 (PMC5298260; doi:10.1371/journal.pone.0171162)
Supplement: S10 Fig — (DOCX) [file pone.0171162.s011.docx]

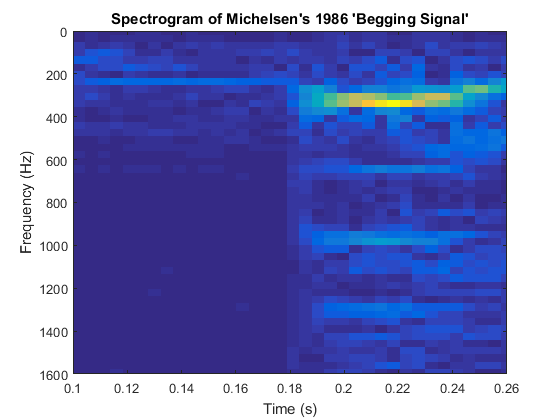


**S10 Fig. Spectrogram of Michelsen’s [21] begging signal** - after it has been processed as shown in Figure S9.

To compare the signal we are detecting with the one recognised as a “begging signal” by previous authors, a Matlab® extraction of the “begging signal” published in Michelsen’s [21] 1986 study was undertaken (S9 Fig). After transforming the velocity measurement into acceleration, the spectrogram of which (S10 Fig) shows a fundamental frequency of 320Hz with upper harmonics at twice, 3x and 4x the fundamental with the second harmonic being the next-most prominent peak. With a time duration of 80ms, his signal matches remarkably well with the thousands of this signals that were detected and analysed with our software.
