# Supplementary material for: Long-term trends in the honeybee ‘whooping signal’ revealed by automated detection
Source: PLoS One. 2017 Feb 8;12(2):e0171162. doi: 10.1371/journal.pone.0171162 (PMC5298260; doi:10.1371/journal.pone.0171162)
Supplement: S11 Fig — a—Fundamental frequency distribution; b–The averaged spectrum of whooping signals with a specific amplitude displayed in descending order from highest (12 a.u.) to lowest (0 a.u.) amplitude Colour codes the measured amplitude in arbitrary units; c–Temporal histograms of whooping signals of a specific amplitude. Colour codes the likelihood of occurrences; d -Daily histogram of whooping signal fundamental frequencies. (DOCX) [file pone.0171162.s012.docx]

*
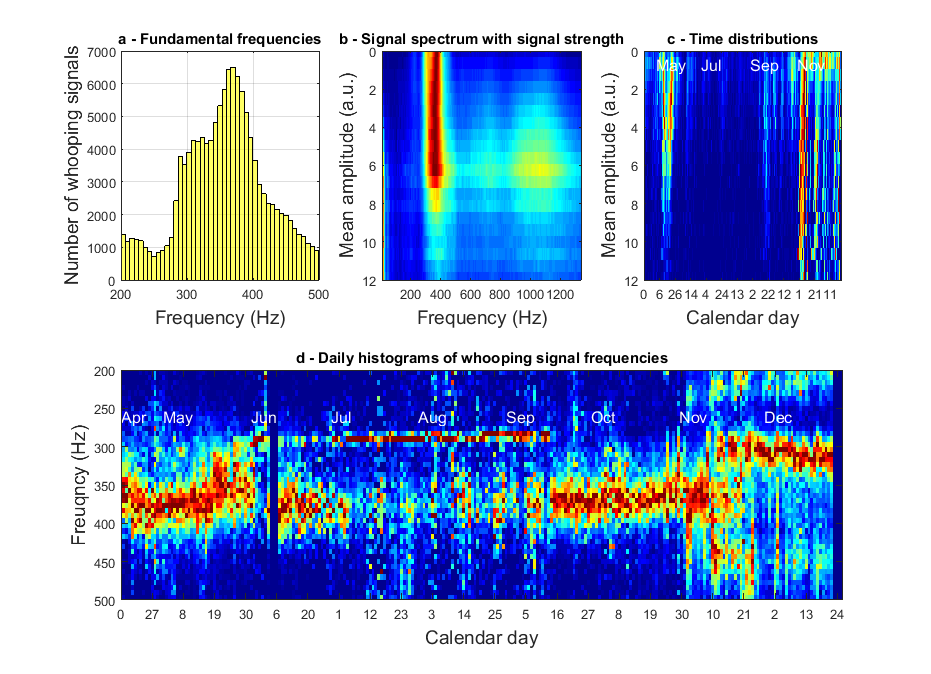
*

Frequency (Hz)

**S11 Fig. Distribution analysis for data coming from the peripheral accelerometer.** **a** - Fundamental frequency distribution; **b** – The averaged spectrum of whooping signals with a specific amplitude displayed in descending order from highest (12 a.u.) to lowest (0 a.u.) amplitude Colour codes the measured amplitude in arbitrary units; **c** – Temporal histograms of whooping signals of a specific amplitude. Colour codes the likelihood of occurrences; **d** -Daily histogram of whooping signal fundamental frequencies.
