# Supplementary material for: Long-term trends in the honeybee ‘whooping signal’ revealed by automated detection
Source: PLoS One. 2017 Feb 8;12(2):e0171162. doi: 10.1371/journal.pone.0171162 (PMC5298260; doi:10.1371/journal.pone.0171162)
Supplement: S12 Fig — The daily histograms of whooping signal fundamental frequencies derived from the centre (a) and periphery (b) of the honeycomb. As in Fig 9, the modal daily accelerometer signal amplitude distribution is superimposed with a red line and acceleration axis given on the right hand side in mm/s2. (DOCX) [file pone.0171162.s013.docx]

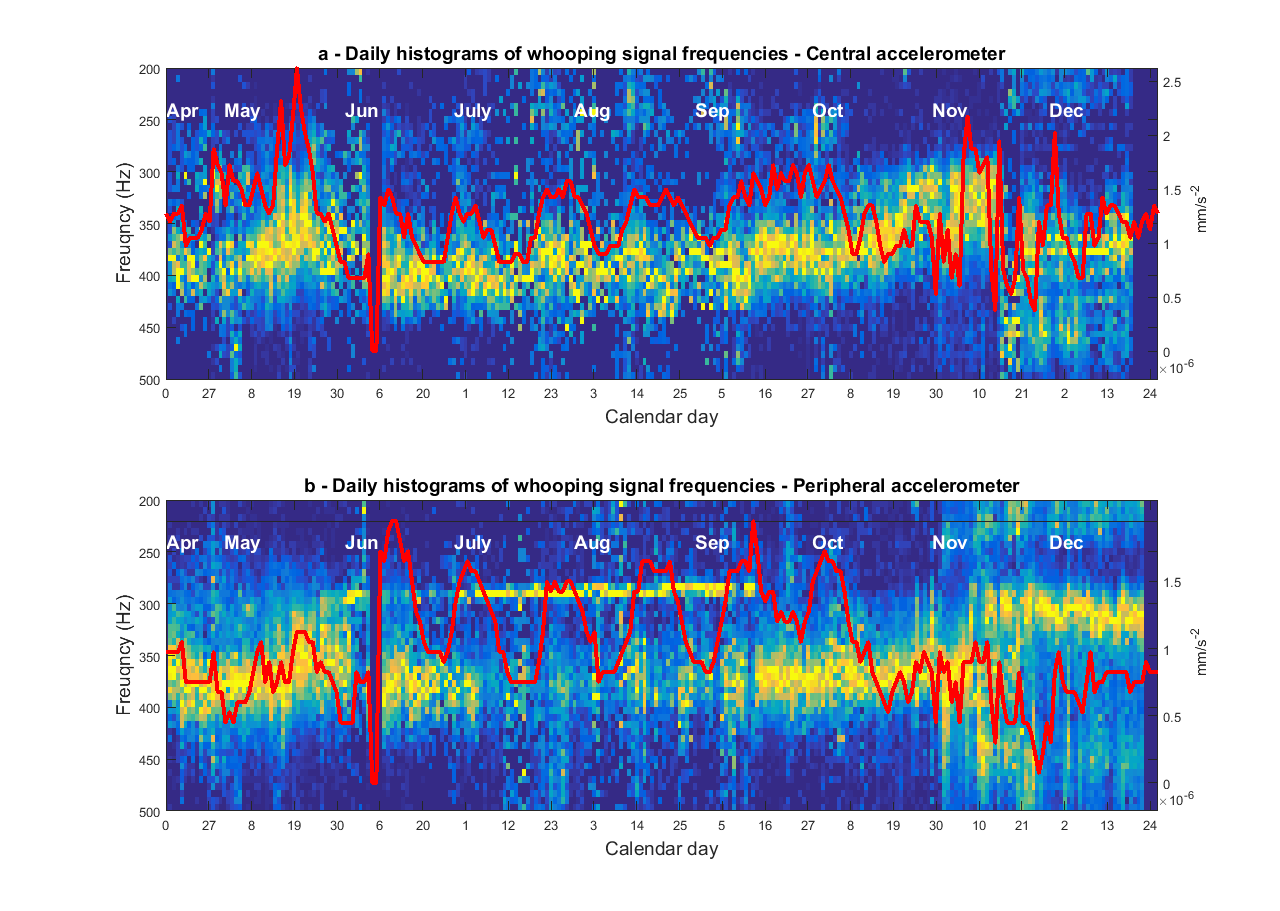


**S12. Fig. The daily histograms of whooping signal fundamental frequencies derived from the centre (a) and periphery (b) of the honeycomb.** As in Fig 9, the modal daily accelerometer signal amplitude distribution is superimposed with a red line and acceleration axis given on the right hand side in mm/s^2^.
