# Supplementary material for: Long-term trends in the honeybee ‘whooping signal’ revealed by automated detection
Source: PLoS One. 2017 Feb 8;12(2):e0171162. doi: 10.1371/journal.pone.0171162 (PMC5298260; doi:10.1371/journal.pone.0171162)
Supplement: S13 Fig — The red line denotes the median, x is the mean, and indents show the confidence intervals at 95%. (DOCX) [file pone.0171162.s014.docx]

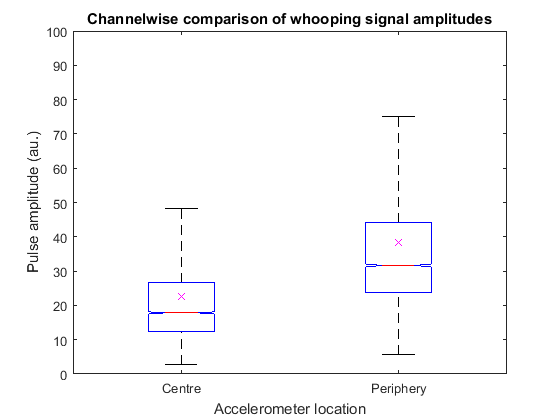


**S13 Fig. Comparison of the peak amplitude of all whooping signals recorded on the central and peripheral accelerometers.** The red line denotes the median, x is the mean, and indents show the confidence intervals at 95%.
