# Supplementary material for: Long-term trends in the honeybee ‘whooping signal’ revealed by automated detection
Source: PLoS One. 2017 Feb 8;12(2):e0171162. doi: 10.1371/journal.pone.0171162 (PMC5298260; doi:10.1371/journal.pone.0171162)
Supplement: S14 Fig — Whooping signals spectra of similar fundamental frequencies were averaged and stacked from left to right. The amplitude is displayed in a logarithmic scale with yellow denoting high amplitude and dark blue denoting low amplitude. (DOCX) [file pone.0171162.s015.docx]

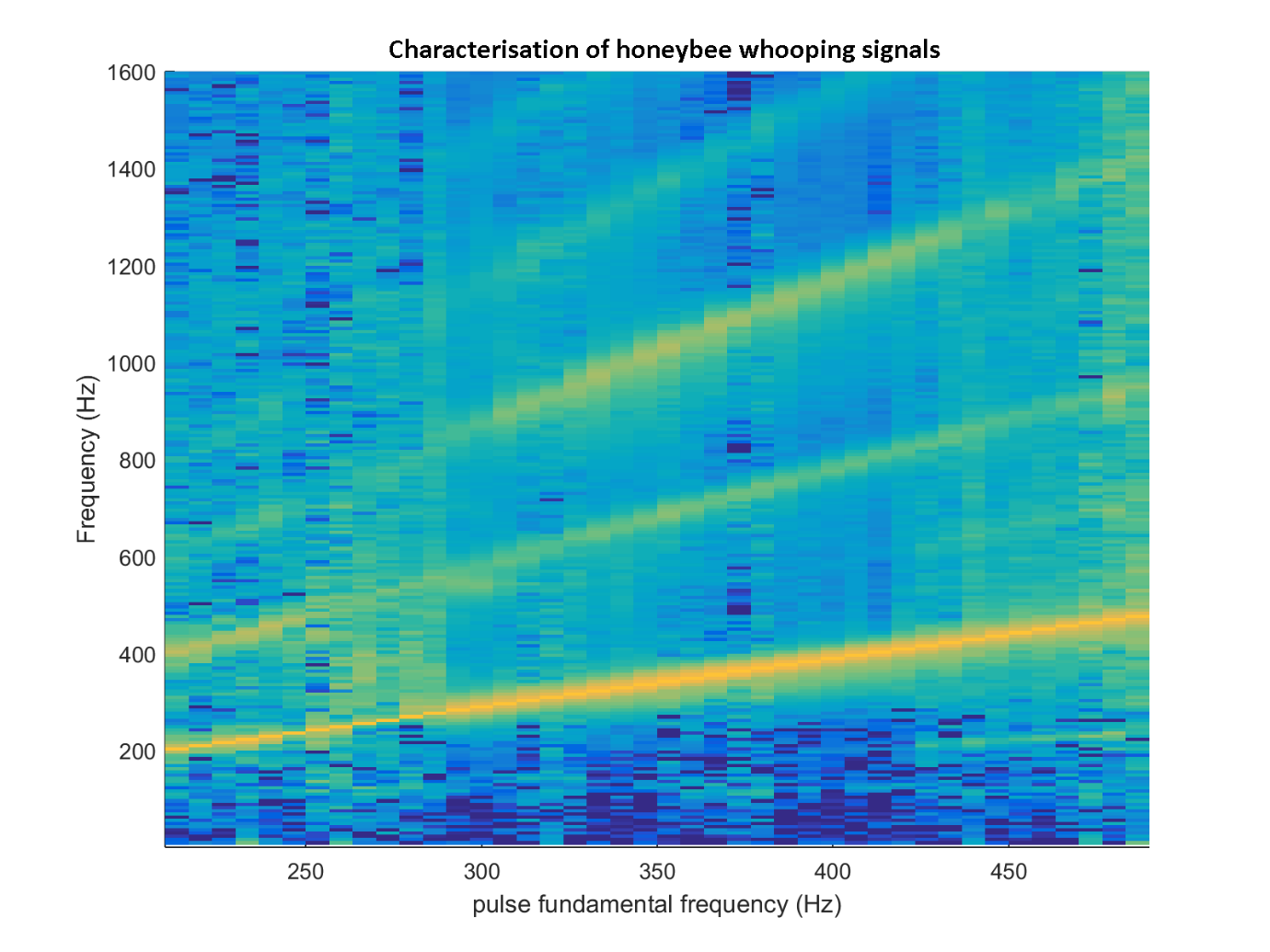


**S14 Fig. Extensive characterisation of honeybee whooping signals on the French dataset.** Whooping signals spectra of similar fundamental frequencies were averaged and stacked from left to right. The amplitude is displayed in a logarithmic scale with yellow denoting high amplitude and dark blue denoting low amplitude*.*

The signal to noise ratio varies substantially along the horizontal axis of this plot, as the number of whooping signals found within a specific bandwidth will vary following the distribution shown in S11d fig. Nevertheless, the evolution of the fundamental frequency and its harmonics can be tracked extremely well over the entire range of frequencies spanning from 200 to 500Hz.
