# Supplementary figures and images for: Long-term trends in the honeybee ‘whooping signal’ revealed by automated detection
Source: PLoS One. 2017 Feb 8;12(2):e0171162. doi: 10.1371/journal.pone.0171162 (PMC5298260; doi:10.1371/journal.pone.0171162)

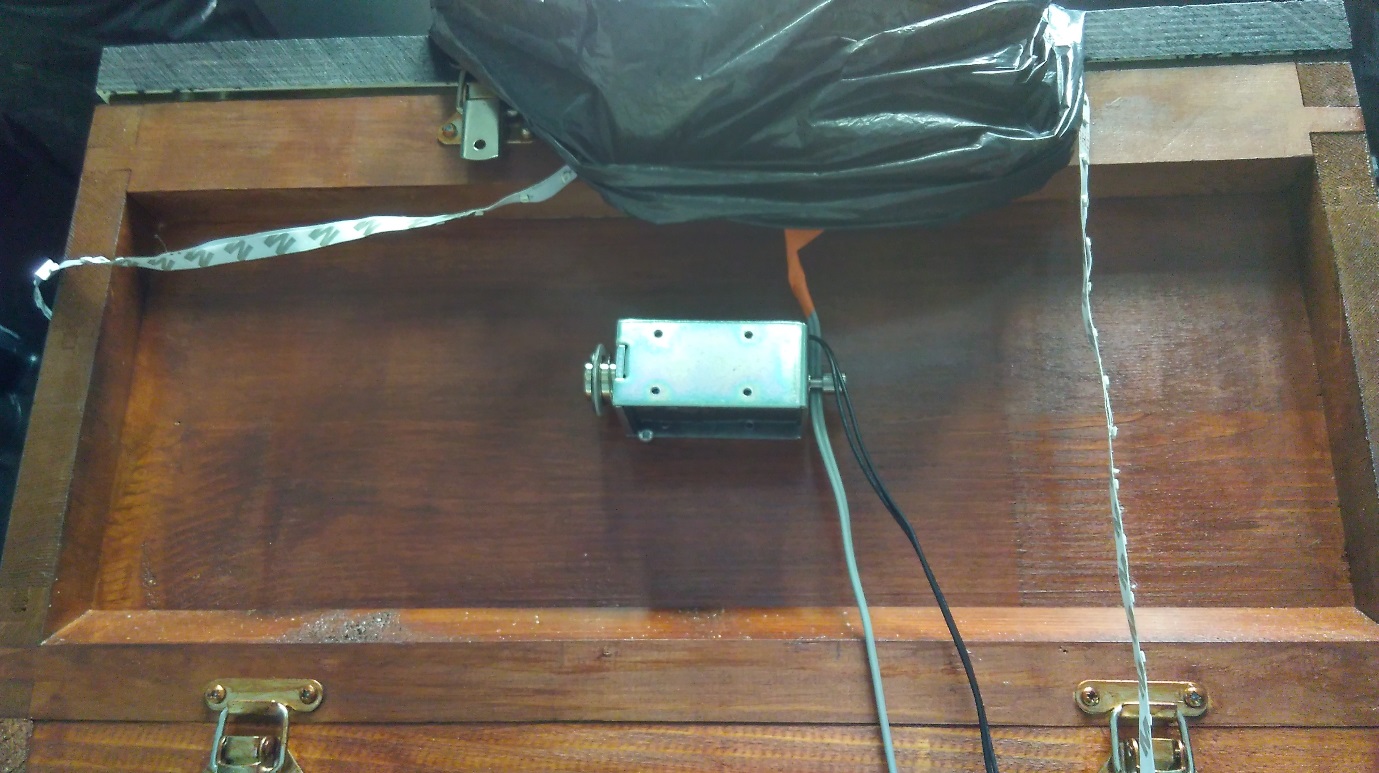


**S15 Fig. Electromagnetic coil secured laterally to the brood box of the observation hive.**

Supplement: S15 Fig — (DOCX) [file pone.0171162.s016.docx]
